# COVID-19: molecular targets, drug repurposing and new avenues for drug discovery

**DOI:** 10.1590/0074-02760200254

**Published:** 2020-10-02

**Authors:** Mario Roberto Senger, Tereza Cristina Santos Evangelista, Rafael Ferreira Dantas, Marcos Vinicius da Silva Santana, Luiz Carlos Saramago Gonçalves, Lauro Ribeiro de Souza Neto, Sabrina Baptista Ferreira, Floriano Paes Silva-Junior

**Affiliations:** 1 Fundação Oswaldo Cruz-Fiocruz, Instituto Oswaldo Cruz, Laboratório de Bioquímica Experimental e Computacional de Fármacos, Rio de Janeiro, RJ, Brasil; 2 Universidade Federal do Rio de Janeiro, Instituto de Química, Laboratório de Síntese Orgânica e Prospecção Biológica, Rio de Janeiro, RJ, Brasil

**Keywords:** COVID-19, SARS-CoV-2, drug repurposing, drug discovery, replication cycle, drug targets

## Abstract

Coronavirus disease 2019 (COVID-19) caused by the severe acute respiratory syndrome coronavirus 2 (SARS-CoV-2) is a highly contagious infection that may break the healthcare system of several countries. Here, we aimed at presenting a critical view of ongoing drug repurposing efforts for COVID-19 as well as discussing opportunities for development of new treatments based on current knowledge of the mechanism of infection and potential targets within. Finally, we also discuss patent protection issues, cost effectiveness and scalability of synthetic routes for some of the most studied repurposing candidates since these are key aspects to meet global demand for COVID-19 treatment.

Epidemiology

Coronavirus disease 2019 (COVID-19) caused by the severe acute respiratory syndrome coronavirus 2 (SARS-CoV-2) is a highly contagious disease that may break the healthcare system of several countries. On January 22, the numbers of confirmed COVID-19 cases were 580 but, at the present date (August 5), these numbers increased to 18.3 million worldwide.^1^ The transmission can occur among humans via oral and nasal respiratory droplets and contact with contaminated surfaces.^2^ For instance, droplets in aerosol from a cough can spread 4 to 5 m and a sneeze can spread droplets up to 8 m away.^3^

Recently the aerodynamic nature of SARS-CoV-2 was also investigated in two Wuhan hospitals in China.^4^ The results showed that the viral RNA level in ventilated patient wards was low because of segregation and high air exchange rate. Conversely, in toilets, which were not ventilated, and medical staff areas had elevated concentrations of airborne viral RNA. After rigorous sanitisation procedures these levels were reduced. Therefore the authors showed that room ventilation, disinfection and sanitisation are important measures to be implemented in hospitals.^4^ Furthermore, SARS-CoV-2 was detected in wastewater in many countries around the world.^5^ ^,^ ^6^ ^,^ ^7^

At the moment, the prevention aimed at reducing transmission in the community is the best alternative, indicating that enhanced public health interventions, including social distancing, use of masks and movement restrictions should be implemented to bring the COVID-19 pandemic under control. Aggressive isolation measures including travel restriction in China, have successfully led to a progressive reduction of COVID-19 cases.^8^ Kissler and colleagues, simulated the relaxation of the protective measures using a post-pandemic mathematical model.^9^ The authors demonstrated that social isolation will be necessary at the best scenario until 2021, and reaffirmed the need and importance of maintaining social isolation. They also suggested that in the absence of such restrictions, the pandemic could last until 2024. Thus, in this context, the search for new medicines available to combat this disease is urgent.

Etiological agent

Coronaviruses are members of the family Coronaviridae that present crown-like spikes on their surface visualised by electron microscopy. The subfamily Coronavirinae contains the four genera Alpha, Beta, Gamma, and Deltacoronavirus. Coronaviruses infect birds (gamma and deltacoronaviruses) and several mammalian species (mainly alpha and betacoronaviruses), including humans.^10^ ^,^ ^11^

Coronaviruses have been isolated from diverse species, including mammals like bats, rodents, bovines, swine, felines, pangolins, horses and others.^12^ ^,^ ^13^ Human coronavirus was first identified in 1966 by Tyrrell and Bynoe.^14^ Nowadays the seven coronaviruses that can infect humans (HCoVs) are classified in alpha coronavirus (229E, NL63) or beta coronavirus [OC43, HKU1, Middle East respiratory syndrome coronavirus (MERS-CoV), severe acute respiratory syndrome coronavirus (SARS-CoV) and more recently the SARS-CoV-2]. 229E, NL63, OC43, and HKU1 can cause upper and lower respiratory tract infection in adults and children. After the 2000s, two epidemic CoVs have arisen in humans: the SARS-CoV and the MERS-CoV which were reported in 2003 and 2012, respectively.^15^ ^,^ ^16^ In 2019, after the first report of novel pneumonia (COVID-19) in Wuhan, China, the seventh hCoV was described. It also causes severe acute respiratory syndrome (therefore called SARS-CoV-2) and spreads very quickly worldwide .

Coronavirus are single-stranded positive-sense RNA viruses and their genome size is approximately 30 kb, which encodes some important structural proteins.^17^ The spike (S) glycoprotein is a well characterised protein that mediates coronavirus entry into host cells via fusion of the viral and cellular membranes through a pre to post fusion conformation transition.^18^ The S protein S1-S2 subunits bind to cellular receptors that vary according to the coronavirus species: angiotensin-converting enzyme 2 (ACE2) in SARS-CoV, SARS-CoV-2 and HCoV-NL63; and dipeptidyl peptidase 4 (DPP4) and aminopeptidase N (APN) in MERS or others alphacoronaviruses like TGEV (porcine transmissible gastroenteritis coronavirus) and porcine respiratory coronavirus (PRCH).^18^ ^,^ ^19^ ^,^ ^20^

Other structural proteins are mandatory to assemble the complete viral particle like nucleocapsid protein (N), membrane protein (M) and the envelope protein (E). Furthermore, they can be involved in other processes like morphogenesis, envelope formation, budding or pathogenesis.^17^ ^,^ ^21^ ^,^ ^22^

By genomic sequencing analysis of other coronavirus strains and SARS-CoV-2, Andersen and collaborators demonstrated that SARS-CoV-2 has mutations resulting in six different amino acids at the receptor-binding domain (RBD) that appears to be optimised for binding to the human receptor ACE2.^23^ They also showed that the gene encoding the Spike protein has an insertion of 12 nucleotides giving it a polybasic (furin) cleavage site at the S1-S2. In this way, the high-affinity of the SARS-CoV-2 spike protein to the human ACE2 is a consequence of natural selection on a human or human-like ACE2. They suggest some possibilities to explain that: emergence in an animal host before zoonotic transfer; natural selection in humans following zoonotic transfer; or natural selection during the passage.^23^ Other researchers did a phylogenetic analysis of 160 genomes of SARS-CoV-2.^24^ They showed 3 important variations in the composition of amino acids that allowed them to classify into different groups. Group A has two subclusters that are distinguished by the synonymous mutation T29095C. While B is derived from A by two mutations T8782C and C28144T, type C differs from its parent type B by mutation of G26144T. The A and C types are found more often outside East Asia, in Europeans and Americans. While the B type is the most common type in East Asia.^24^

Clinical aspects of pathology

In December 2019, COVID-19 was initially reported as a new viral pneumonia, due to the clinical characteristics of the large number of cases that emerged in Wuhan, China.^25^ ^,^ ^26^ SARS-CoV-2 typically causes respiratory sickness, the major clinical characteristics observed in infected patients are high fever, dry cough and dyspnea (shortness of breath or difficulty in breathing). Minor symptoms include headache, diarrhea, nausea, vomiting, loss of smell and taste.^27^ This clinical condition can progress to moderate or severe pneumonia.^28^ In this case, first there is an accumulation of macrophages in alveoli, followed by release of cytokines and accumulation of fluids. Neutrophils can also be recruited by the immune system leading to the destruction of type I and type II alveolar epithelial cells causing a collapse of the alveoli function and consequently the acute respiratory distress syndrome (ARDS). In the severe condition, with an increase of the inflammation, the protein rich fluid from lungs enter in the bloodstream causing the systemic inflammatory syndrome (SIRS).^29^ ^,^ ^30^ These complicating factors can lead to a multi-organ failure and septic shock, causing patient death.

Furthermore, some pre-existing conditions can enhance the risk to develop the severe form of the disease, including age over 60 years and a history of chronic diseases like chronic lung disease, asthma, heart diseases, immunosuppressed patients, cancer, diabetes or chronic kidney disease.^31^ ^,^ ^32^ ^,^ ^33^ ^,^ ^34^ ^,^ ^35^ Finally, the vast majority of people have mild symptoms or are asymptomatic, which is a big problem because they can also transmit the virus to the non-infected population.^36^

Drug repositioning

Drug repositioning, repurposing, reprofiling or re-tasking is the evaluation of existing drugs for new therapeutic purposes.^37^ A candidate drug (investigational or approved) for repurposing efforts already has a known safety and toxicity profile, based on at least successful Phase I or Phase II clinical trials.^38^ Considering the whole process, costs of bringing a repurposed drug to the market have been estimated to be ten times lower and the time is shortened by around a half, compared with a new drug.^38^ Even though the clinical phase III and regulatory aspects remain similar for developing a new drug, drug repurposing possesses many advantages over developing a new drug from scratch: the reduced time and financial investment for development, the lower risk of failure and a consolidated pharmaceutical supply chain for production and distribution to the patients that effectively need treatment.^39^

Emerging or reemerging viruses pose major public health concerns globally.^40^ For several pathogenic viruses, considerable efforts have focused on vaccine development and other therapies, like transfusion of convalescent plasma.^41^ ^,^ ^42^ However, during pandemics infected individuals need urgently to be treated on a large scale. A medicine armamentarium for the COVID-19 outbreak is needed immediately and drug repurposing could be one of the best strategies to deal with this pandemic.^43^ ^,^ ^44^ Computational and experimental approaches can be used, alone or combined, to achieve a more holistic point of view and increased chance of success in drug repurposing.

In the following topic, we will review SARS-CoV-2 structure and mechanism of infection in order to discuss molecular targets from the virus or its human host that are being considered for drug repurposing and perhaps future development of new drugs. Ongoing drug repurposing efforts will be described in more details later in this article, along with some clinical trials that have been carried out so far for COVID-19 treatment. Finally, as treatment availability is of utmost importance when dealing with a pandemic, we bring a discussion on patent protection and ease of large-scale production of some of the drugs that are more advanced in clinical studies.

SARS-CoV-2: structure, mechanism of infection and drug targets

*SARS-CoV-2 structure* - Electron microscopy imaging of SARS-CoV-2 virions indicates that they have a spherical or pleomorphic shape, with diameters ranging from 60 to 140 nm, showing prominent spikes of 9-12 nm in their surfaces that resemble a solar corona, hence the name “coronavirus”.^45^ ^,^ ^46^ ^,^ ^47^ ^,^ ^48^ SARS-CoV-2 is an enveloped virus with a single-stranded positive sense (5’-3’) RNA (+ssRNA) (~ 30 kb) containing a 5’-cap structure and a 3’-poly-A tail.^49^ ^,^ ^50^ Its genomic RNA (gRNA) has a variable number of open reading frames (ORFs) that are predicted to encode 16 non-structural (Nsp), 4 structural and several accessory proteins (Fig. 1).^26^ ^,^ ^51^ ^,^ ^52^ ^,^ ^53^ ^,^ ^54^ ORF1a and ORF1b represent more than 2/3 of the whole length of gRNA, and encode two polyproteins: pp1a (440-500 kDa) and pp1ab (740-810 kDa).^53^ ^,^ ^55^ The polyprotein pp1a is translated from ORF1a while pp1ab from ORF1a/ORF1b using a -1 ribosomal frameshift mechanism that occurs near the 3’ end of ORF1a which allows continued translation of ORF1b.^53^ Together, pp1a and pp1ab originate all Nsps (1-16), such as M^pro^ (Nsp5) protease and RdRp (Nsp12) RNA polymerase, which form viral replicase/transcriptase complexes (RTCs), and are encapsulated in double-layered vesicles originated from the endoplasmic reticulum (ER).^56^ ^,^ ^57^ ^,^ ^58^

**Fig. 1:**
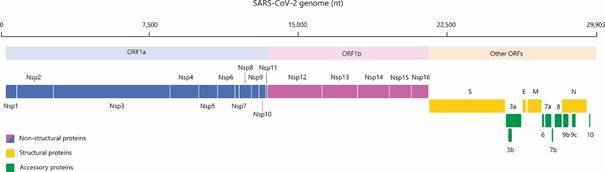
genomic structure of severe acute respiratory syndrome coronavirus 2 (SARS-CoV-2) and its encoded proteins. Together, open reading frames (ORFs) 1a and 1b are translated into all 16 non-structural proteins (Nsp1-16) while the remaining ORFs encode the structural (S, E, M, N) and accessory proteins (here represented as nine individual proteins). Adapted from Gordon et al.^54^

The ORFs near the 3’ end of the gRNA encode the structural and accessory proteins of SARS-CoVs.^58^ The first ones have a crucial role in the assembly of viral particles and virus invasion.^56^ ^,^ ^58^ The main structural proteins are named: spike (S), envelope (E), nucleocapsid (N) and membrane (M) proteins. Most of them reside on the virion surface (S, E, M proteins) while N proteins are found in the core of the particle bound to gRNA.^59^ S proteins are essential for virus attachment and entry into the host cells, tissue tropism and pathogenesis.^58^ ^,^ ^60^ E proteins exert several roles in virus infection, such as helping in virus assembly and release from infected cells, creating ion channels in cell membranes and suppressing host stress response.^58^ ^,^ ^61^ ^,^ ^62^ N proteins interact with gRNA to form the ribonucleoprotein.^56^ ^,^ ^62^ M proteins have a role in virion assembly and in determining the shape of the envelope. They also bind to all other structural proteins promoting, for instance, the stabilisation of N protein-RNA complexes.^56^ ^,^ ^63^

*SARS-CoV-2 mechanism of infection* - At present, the mechanisms that underlie SARS-CoV-2 infection have not been directly described. Nonetheless, they seem to be similar to those proposed for other coronaviruses.^58^ In one proposal, virus infection starts with the binding of its S proteins to host receptor ACE2, a membrane protein largely expressed in the lung and small intestine cells (Fig. 2).^44^ ^,^ ^59^ ^,^ ^64^ After attachment, S protein is cleaved by host proteases initiating the fusion of virus and cell membranes that culminates in viral gRNA release into the cytoplasm. This event is proposed to occur through two distinct ways: via plasma membrane (early pathway) or via endosomes (late pathway). In the early pathway, S protein is cleaved by host plasma membrane proteases (e. g., TMPRSS2) while in the late pathway by endosomal proteases (e. g., cathepsin L). The route taken by the virus to enter the cell appears to be dependent on the availability of these proteases.^59^ ^,^ ^64^ ^,^ ^65^

**Fig. 2:**
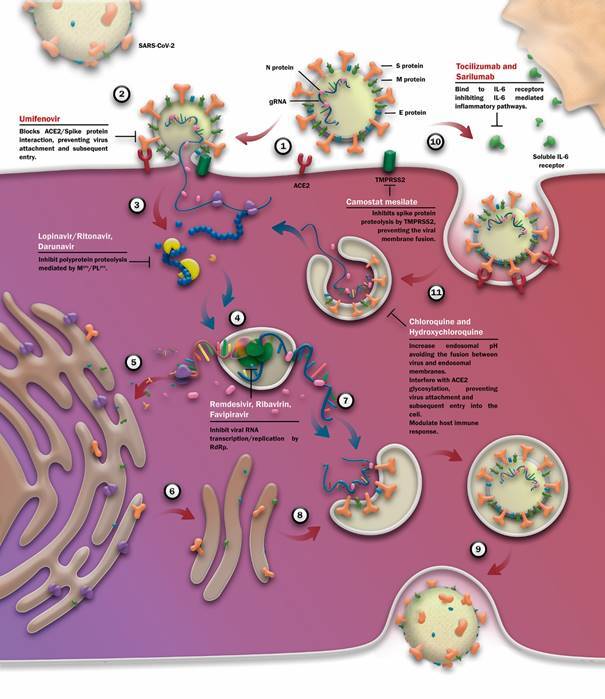
severe acute respiratory syndrome coronavirus 2 (SARS-CoV-2) replication cycle and potential targets for drug repurposing. (1) Virus infection initiates with the binding of virus S proteins to the ACE2 cellular receptors. After attachment, the virus may enter the cell through two distinct mechanisms: early and late pathways. (2) In the early pathway the genomic ssRNA (gRNA) is liberated into the cytoplasm after the fusion between viral and cell cytoplasmic membranes, an event triggered by membrane proteases (e. g., TMPRSS2). (3) The gRNA is immediately translated into two polyproteins that undergo proteolytic cleavage giving rise to all nonstructural proteins (Nsps). (4) The Nsps form the replication-transcription complexes (RTCs) where the gRNA (blue ribbon) is replicated and the subgenomic RNAs (red ribbon) are transcribed. (5) The subgenomic RNAs are translated into viral structural and accessory proteins in the cytosol. (6) Upon translation, E, M and S structural proteins are inserted into ER and follow the secretory pathway to the ER-Golgi intermediate compartment (ERGIC). (7) Meanwhile, a copy of the gRNA binds to N proteins in the cytoplasm forming the nucleocapsid, which is transported to the ERGIC. (8) Virion assembly in the ERGIC. (9) The new virion travels through the cytoplasm inside a vesicle and leaves the cell by exocytosis. (10) Alternatively, in the late pathway, the virus can undergo endocytosis to initiate the infection. (11) The virion membrane merges with the endosome membrane after S protein proteolysis by endosomal proteases (e. g., cathepsin L), allowing the gRNA to be released into the cytoplasm. From this point on, the cycle follows the same pathway described in 3-9 steps. The red arrows indicate the general replication pathway and the blue arrows indicate the gRNA movement through the cycle. Some viral and host proteins have been explored as potential targets for drug repurposing. Some of these drug candidates are shown with their site of action indicated in the cycle.^44^ ^,^ ^59^ ^,^ ^66^ ^,^ ^67^

Once in the cytoplasm, gRNA is readily translated into viral polyproteins (pp1a/pp1ab), which are cleaved into the individual Nsps that compose the RTCs (Fig. 2). These complexes recognise transcriptional regulator sequences in gRNA and begin to transcribe a series of subgenomic RNAs that encode structural and accessory proteins, otherwise the whole gRNA is replicated.^57^ ^,^ ^58^ ^,^ ^59^ Upon translation, S, E and M structural proteins are driven to the ER-Golgi intermediate compartment (ERGIC) where S proteins go through post-translational modifications (e. g., proteolysis, N-glycosylation). In parallel, a copy of the gRNA and N proteins bind in the cytoplasm to form the nucleocapsid and move into the ERGIC. In this compartment, nucleocapsid and the other viral proteins are assembled into a virion which travels through the cytoplasm inside a vesicle and leaves the cell by exocytosis.^48^ ^,^ ^56^ ^,^ ^59^

*Candidate drug targets* - In the search for a treatment for COVID-19, several viral and host molecular proteins have been explored as potential drug targets. Overall, they participate in key events of the virus infection cycle, such as cell entry and replication, as well in host metabolic pathways and immune response. In the following topics we will address in more details some of these targets. Drugs under investigation for blocking the main steps of the SARS-Cov-2 virus replication cycle are indicated in Fig. 2.

*Virus targets* - During SARS-CoV-2 gRNA translation, two proteases, namely M^pro^ and PL^pro^, act in concert to cleave and release from pp1a/pp1ab the 16 Nsps that compose the RTC.^58^ Therefore, these proteases are essential for virus replication and represent useful targets for therapeutic intervention. Recently, SARS-CoV-2 M^pro^ and PL^pro^ had their 3D structures published - PDB 6LU7, 2.16 Å and PDB 6W9C, 2.70 Å, respectively - which make them particularly useful for computational structure-based drug design methods.^68^

3-chymotrypsin-like protein (synonyms: coronavirus main protease, M^pro^, 3CL^pro^) of SARS-CoV-2 is a 33.8 kDa homodimeric protein (306 aa) that belongs to the cysteine protease class (possibly from C30 family and PA clan).^68^ ^,^ ^69^ This enzyme catalyses the hydrolysis of peptide bonds of polyproteins (possibly E.C. 3.4.22.69) at sites whose amino acid sequences generally follow the pattern Leu-Gln* (Ser, Ala, Gly) (*marks the cleavage site).^70^ ^,^ ^71^ M^pro^ is part of pp1a/pp1ab polyproteins (Nsp5).^57^ During polyproteins translation, M^pro^ suffers autolytic cleavage and is released from its polyprotein precursor, reaching a mature state that cleaves pp1a/pp1ab at no less than 11 sites downstream of the Nsp4 coding region.^57^ ^,^ ^68^ ^,^ ^70^ ^,^ ^72^ Each protomer of SARS-CoV-2 M^pro^ is divided into three domains: chymotrypsin and picornavirus 3C protease-like I and II domains, composed by antiparallel β-barrel structures, and domain III which contains five α-helices arranged into an antiparallel globular cluster responsible for protease dimerisation (Fig. 3A). Domains II and III are connected by a long loop, where lies a cleft that serves as a substrate binding site and where catalysis occurs using the Cys^145^-His^41^ dyad (Fig. 3B).^68^ ^,^ ^70^ ^,^ ^73^ M^pro^ has been widely explored in drug discovery campaigns using experimental and/or computational approaches.^68^ ^,^ ^73^ ^,^ ^74^ ^,^ ^75^ ^,^ ^76^ ^,^ ^77^ Moreover, M^pro^ has no human homologue, which reduces the chances of toxic effects of a given inhibitor.^68^ Potential SARS-CoV-2 M^pro^ inhibitors include FDA-approved antivirals, such as inhibitors of HIV-1 [e. g., lopinavir (1) /ritonavir (2)] and HCV [e.g., boceprevir (3)] proteases, as well as antineoplastic [e.g. carmofur (4)] and antibacterial [e.g., doxycycline (5)] drugs.^73^ ^,^ ^78^ ^,^ ^79^ ^,^ ^80^ ^,^ ^81^ ^,^ ^82^ Chemical structures for compounds 1-14 are shown in Fig. 4.

**Fig. 3:**
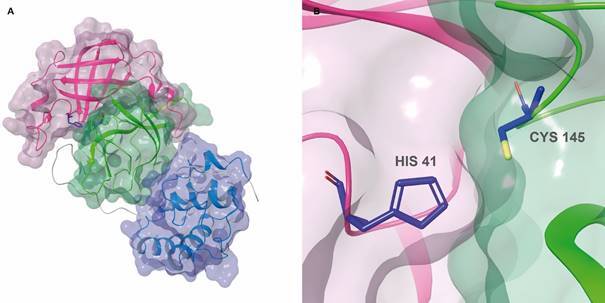
severe acute respiratory syndrome coronavirus 2 (SARS-CoV-2) main protease (M^pro^) 3D structure (PDB 6LU7, 2.16 Å). (A) Transparent VdW surface and ribbon representation of M^pro^, coloured by its three domains: domain I (pink), domain II (green) and domain III (blue). (B) The catalytic pair formed by Cys145 and His41 (CPK colours with dark blue carbons). Images created using Maestro, release 2020-1 (Maestro, Schrödinger, LLC, New York, NY, 2020).

**Fig. 4:**
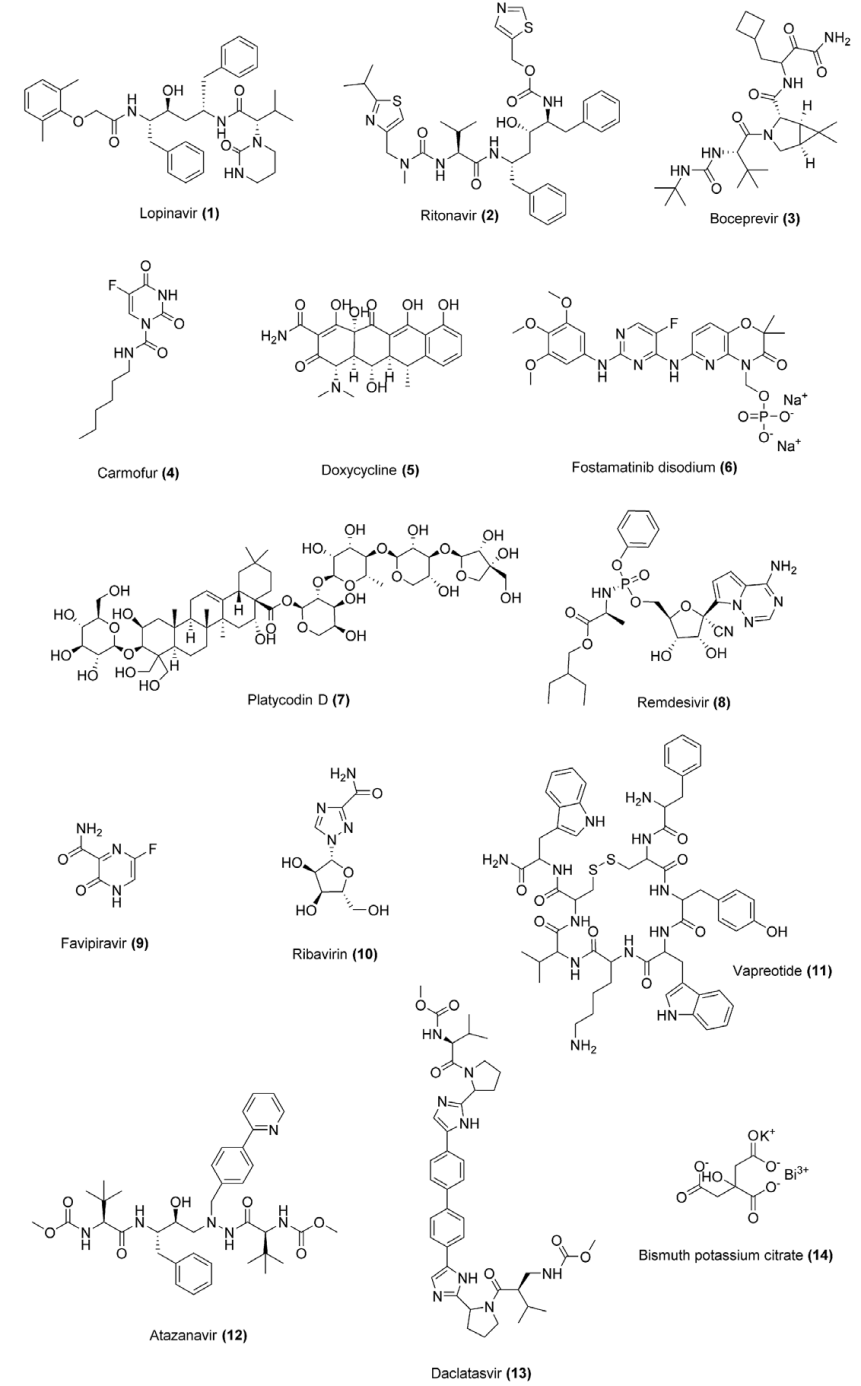
chemical structures for drug repurposing candidates and compounds 1-14 under investigation against coronavirus disease 2019 (COVID-19).

Nsp3 is the largest protein encoded by CoVs (~200 kDa). In SARS-CoVs it has 16 domains which include a papain-like proteolytic enzyme from the cysteine protease class (C16 family and CA clan for SARS-CoV).^57^ ^,^ ^69^ ^,^ ^83^ PL^pro^ is composed of a catalytic domain, an extended right-handed thumb-palm-finger structure with a Cys-His-Asp catalytic triad, and a ubiquitin-like domain (Ubl) (Fig. 5A). The catalytic Cys is located in the thumb subdomain while His and Asp in the palm subdomain (Fig. 5B). PL^pro^ catalyses the hydrolysis reaction of peptide bonds of pp1a/pp1ab at three sites (Nsp1/Nsp2, Nsp2/Nsp3 and Nsp3/Nsp4) that share the XLXGG* pattern (*represents the cleavage site).^57^ ^,^ ^71^ ^,^ ^83^ ^,^ ^84^ This activity is responsible for Nsp3 release from polyproteins. PL^pro^ also recognises and hydrolyses ubiquitin and ISG15 from cellular proteins.^83^ ^,^ ^84^ The deubiquitination and deISGylation activities are proposed to modulate the post-translational modifications of signaling molecules that trigger innate immune response of the host.^85^ These functions are pivotal for virus infection justifying the search for SARS-CoVs PL^pro^ inhibitors.^84^ ^,^ ^86^ ^,^ ^87^ ^,^ ^88^ ^,^ ^89^ ^,^ ^90^ Putative inhibitors of SARS-CoV-2 PL^pro^ include FDA-approved drugs such as fostamatinib disodium (6) (a tyrosine kinase inhibitor used in the treatment of chronic immune thrombocytopenia) and natural products [e.g., platycodin D (7)].^89^ ^,^ ^91^

**Fig. 5:**
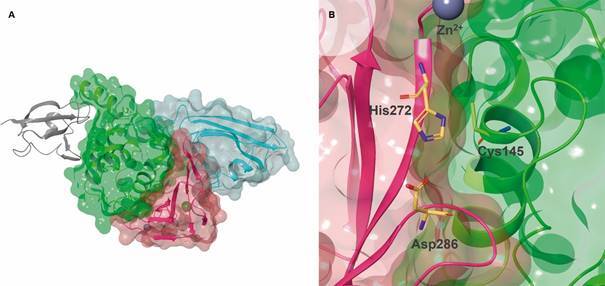
tertiary structure and catalytic site of severe acute respiratory syndrome coronavirus 2 (SARS-CoV-2) PL^pro^ (PDB 6W9C, 2.7 Å). (A) The monomer is represented with transparent VdW surface and opaque ribbons, coloured by domains: finger (cyan), palm (pink) and thumb (green). The Ubl domain is represented in dark gray ribbon. (B) Amino acid residues of the catalytic triad (Cys145, His272 and Asp286) are represented in sticks coloured by CPK (carbons in yellow). The Cys145 is located in the thumb domain (green) while His272 and Asp286 are located in the palm domain (pink). Images created using Maestro, release 2020-1 (Maestro, Schrödinger, LLC, New York, NY, 2020).

RNA-dependent RNA polymerase (RdRp, Nsp12) is an essential protein responsible for RNA synthesis during viral RNA transcription and replication cycles.^89^ As described for SARS-CoV, the RNA polymerase activity of SAR-CoV-2 RdRp (804 aa) also seems to require the binding of Nsp7 and Nsp8 cofactors to enhance RdRp binding and processivity.^92^ ^,^ ^93^ The overall RdRp structure has a “right hand” RdRp domain, composed by three subdomains (palm, fingers and thumb), and a nidovirus-unique N-terminal extension domain that forms a nidovirus RdRp-associated nucleotidyltransferase (NiRAN) structure. These domains are connected by an interface domain. Additionally, this protein also has a N-terminal β-hairpin (Fig. 6A). The active site of RdRp contains conserved polymerase motifs located in the palm subdomain and its configuration is similar to other RNA polymerases.^94^ RdRp substrates, RNA template/primer and nucleotide triphosphate (NTPs), access the catalytic centre through the template and NTP entry paths, respectively, while the product-template hybrid is released through the RNA exit path.^92^ ^,^ ^94^ SARS-CoV-2 RdRp shares 96% identity in amino acid sequence with SARS-CoV protein.^93^ Moreover, the accessory proteins also have a high degree of amino acid sequence identity between these viruses: 98.1 % for Nsp7 and 97.5 % for Nsp8 - sequences obtained from PDB 7BV2 (2.5 Å) and PDB 6NUR (3.1 Å) entries.^92^ ^,^ ^95^ Therefore, it is reasonable to suggest that SARS-CoV RdRp inhibitors may also bind to the homologous enzyme from SARS-CoV-2, as demonstrated for remdesivir (8) (Fig. 6B), an adenosine triphosphate analog.^92^ ^,^ ^93^ ^,^ ^96^ Other potential inhibitors include clinically available drugs, such as favipiravir (9), a purine nucleic acid analog used in the treatment of influenza, and ribavirin (10), a synthetic guanosine nucleoside indicated for the treatment of hepatitis C virus (HCV) infection.^97^ ^,^ ^98^ The crucial role of RdRp and the lack of a host homolog turns this enzyme into a valuable target for anti-CoVs agents.^93^

**Fig. 6:**
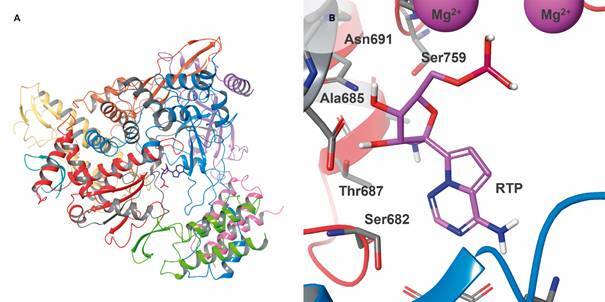
3D structure of severe acute respiratory syndrome coronavirus 2 (SARS-CoV-2) RNA-dependent RNA polymerase (PDB 7BV2, 2.5 Å, RdRp, Nsp12) bound to triphosphate form of remdesivir (RTP) and cofactors Nsp7 (pink) and Nsp8 (purple). (A) Ribbon representation coloured by Nsp12 subdomains: β-hairpin (cyan), NiRAN (yellow), interface (orange), finger (blue), palm (red) and thumb (light green). (B) Binding pose of RTP. The sidechain of all residues (gray) within 5 Å of distance and the RTP molecule (pink) are represented as sticks. Magnesium ions (pink) are represented as space filling spheres. Images created using Maestro, release 2020-1 (Maestro, Schrödinger, LLC, New York, NY, 2020).

Helicase (Nsp13) is a multifunctional protein (predicted to have 596 aa in SARS-CoV-2) essential for SARS-CoVs RNA replication and proliferation.^74^ ^,^ ^99^ SARS-CoV helicase belongs to helicase superfamily 1 (SF1) and can unwind double-stranded DNAs/RNAs (helicase activity) in the 5’-3’ direction, an energy-consuming process that is driven by the hydrolysis of nucleosides triphosphate (NTPase activity).^57^ ^,^ ^100^ ^,^ ^101^ Its structure contains five domains arranged in a triangular pyramid-shape: the triangular base is composed by two “RecA-like” (1A and 2A) and 1B domains whereas the zinc binding domain (ZBD), in the N-terminal, and the stalk domain are oriented towards the apex. The ZBD and 1B domains are connected by the stalk domain (Fig. 7).^101^ The same domain arrangement is predicted to occur in SARS-CoV-2 enzyme.^74^ SARS-CoV helicase has been explored as a potential target for drugs.^99^ ^,^ ^100^ ^,^ ^102^ Recently, some efforts have also been made to predict inhibitors for SARS-CoV-2 helicase.^74^ ^,^ ^103^ ^,^ ^104^ ^,^ ^105^ They include drugs used in the clinics to treat acquired immunodeficiency syndrome (AIDS), such as vapreotide (11) (somatostatin analog) and atazanavir (12) (HIV protease inhibitor), as well anti-HCV protease inhibitors [e.g., daclatasvir (13)] and bismuth salts [e.g., bismuth potassium citrate (14), a compound used in clinical treatment of gastrointestinal diseases].^103^ ^,^ ^104^ ^,^ ^106^

**Fig. 7:**
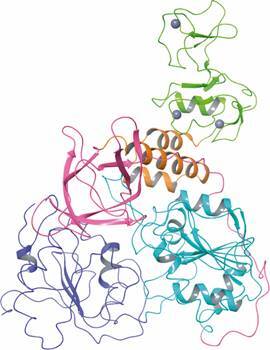
tertiary structure of severe acute respiratory syndrome coronavirus (SARS-CoV) helicase (PDB 6JYT, 2.8 Å, Nsp13). The domains are zinc-binding (ZBD) (light green), stalk (orange), 1B (pink), 1A (cyan) and 2A (blue). The zinc atoms are shown as gray spheres. Image created using Maestro, release 2020-1 (Maestro, Schrödinger, LLC, New York, NY, 2020).

SARS-CoVs replication also requires other enzymatic activities including (guanine-N7)-methyltransferase (N7-MTase), 2’-O-methyltransferase (2’-OMTase) and exoribonuclease (ExoN).^57^ ^,^ ^107^ ^,^ ^108^ The N7-MTase domain at the C-terminus of Nsp14 is responsible for adding a methyl group in the N7 position of RNA 5’-guanosine forming the 5’-cap structure of viral RNAs.^108^ Additionally, Nsp14 also has a catalytic domain in its N-terminus with 3’-5’ ExoN activity, which participates in RNA proofreading mechanism (Fig. 8).^57^ 2’-OMTase (Nsp16) catalyses the methylation reaction at the ribose 2’-O position of the first and second nucleotide of the mRNAs and it is one of the SARS-CoV-2 proteins whose 3D structure has already been resolved (PDB: 6W61, 2.0 Å) (Fig. 9).^109^ Both Nsp14 and 16 use Nsp10 as a cofactor to enhance their 3’-5’ ExoN and 2’-OMTase activities, respectively.^110^ Together, these proteins are responsible for inducing RNA modifications that are essential for its stability and translation as well to avoid the activation of host immune response.^57^ ^,^ ^107^ ^,^ ^109^ Thus, Nsp14 and 16 have been considered as potential targets for anti-SARS agents that may affect their functions in a direct or indirect way (e.g., by blocking Nsp 10 binding).^108^ ^,^ ^109^ ^,^ ^110^ ^,^ ^111^ ^,^ ^112^ ^,^ ^113^ ^,^ ^114^ ^,^ ^115^ Some compounds have been regarded as potential inhibitors of SARS-CoV-2 methyl transferases, such as adenine dinucleoside S-adenosylmethionine analogs [e.g., dinucleoside 13 (15)], predicted for SARS-CoV N7-MTase activity, and some clinically available drugs [e.g., raltegravir (16) - a HIV integrase inhibitor], predicted for SARS-CoV-2 2’-OMTase activity.^112^ ^,^ ^115^ Chemical structures for compounds 15-29 are shown in Fig. 10.

**Fig. 8:**
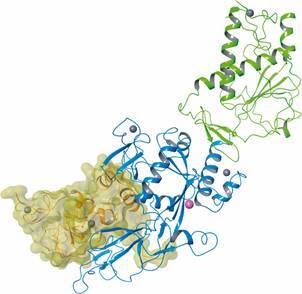
tertiary structure of severe acute respiratory syndrome coronavirus (SARS-CoV) Nsp14 (PDB 5C8S, 3.3 Å) in complex with its Nsp10 cofactor (yellow VdW surface). Nsp14 is represented as ribbons coloured according to its domains: Methyltransferase (MTD, light green) and exoribonuclease (ExoN, blue). Zn2+ and Mg2+ ions are shown as gray and magenta spheres, respectively. The Nsp10 cofactor interacts with the Nsp14 ExoN domain. Image created using Maestro, release 2020-1 (Maestro, Schrödinger, LLC, New York, NY, 2020).

**Fig. 9:**
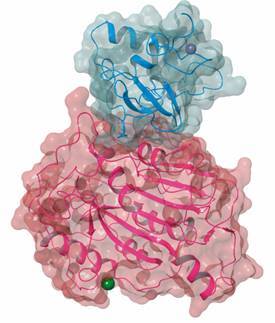
ribbon representation and VdW surface of the severe acute respiratory syndrome coronavirus 2 (SARS-CoV-2) 2’-O-methyltransferase 3D structure (PDB 6W61, 2.0 Å, Nsp16, pink) in complex with its Nsp10 cofactor (light blue). The zinc and chloride ions are represented as gray and green spheres, respectively. Image created using Maestro, release 2020-1 (Maestro, Schrödinger, LLC, New York, NY, 2020).

**Fig. 10:**
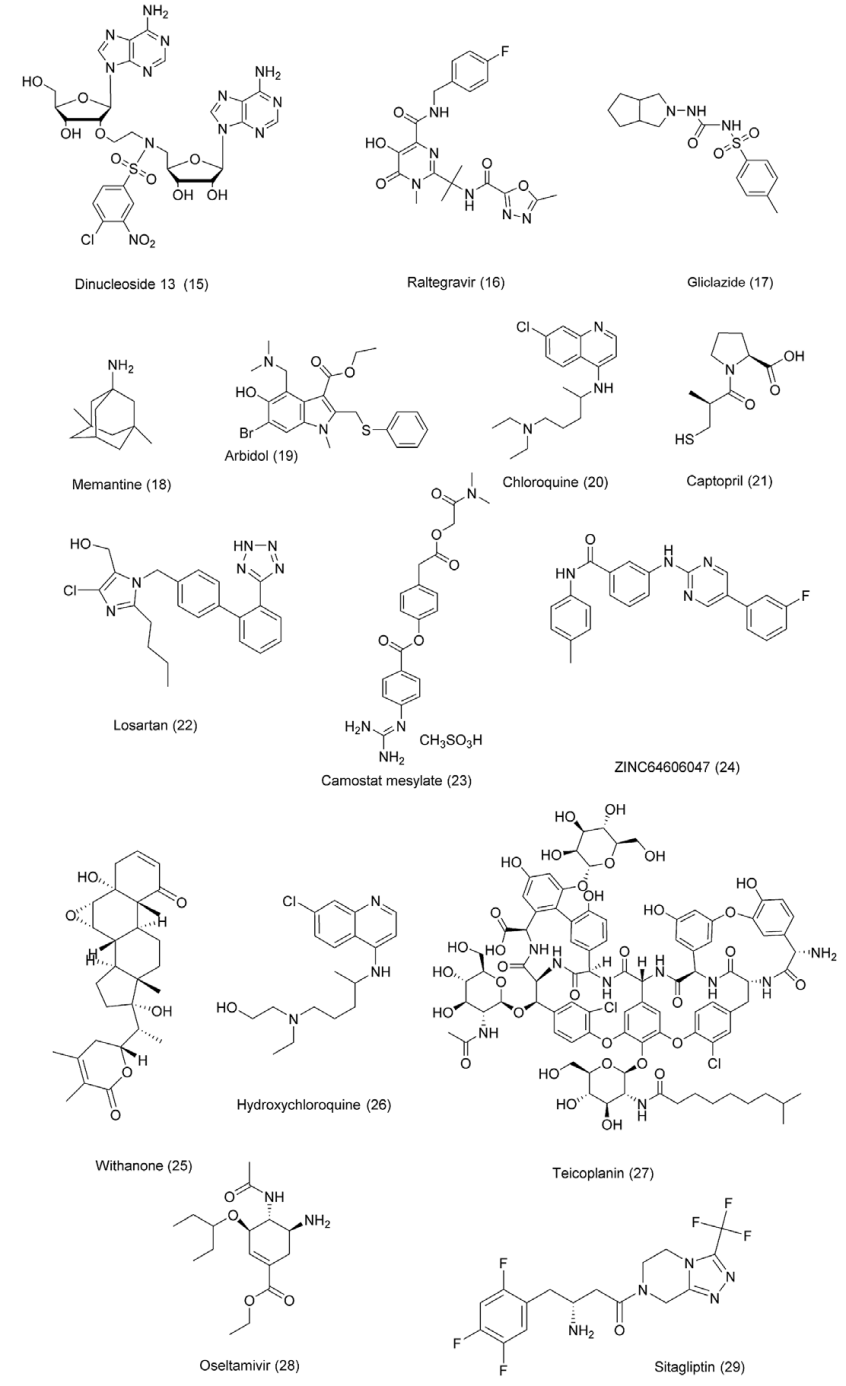
chemical structures for drug repurposing candidates and compounds 15-29 under investigation against coronavirus disease 2019 (COVID-19).

Spike protein (S) is a viral type I transmembrane glycoprotein (~180-200 kDa) responsible for CoVs interaction and invasion of host cells.^59^ ^,^ ^64^ This protein is synthesised as a monomer and suffers post-translational modifications in the ER before becoming a trimeric glycosylated protein.^59^ ^,^ ^116^ Its structure is divided into three main domains: an extracellular, a transmembrane and a short intracellular domain (Fig. 11).^117^ The former protrudes from the surface of the virus particle creating a crown-like halo and contains two functional subunits: S1 (bulbous shape), which binds to cellular receptors (e.g., ACE2), and S2 (stalk shape) that promotes the fusion between cell and virus membranes.^59^ In turn, S1 subunit has two domains: a N-terminal and a C-terminal domain. The latter serves as a RBD for SARS-CoVs being responsible for ACE2 recognition and binding.^59^ ^,^ ^64^ ^,^ ^117^ Apart from S1 and S2, SARS-CoV-2 S protein also has a ganglioside-binding subdomain at the tip of N-terminal domain, which may allow this protein to interact with gangliosides on cells’ surface. In theory, this domain could facilitate virus attachment to the cell and facilitate the contact with ACE2, being a potential site for drug interference.^118^

**Fig. 11:**
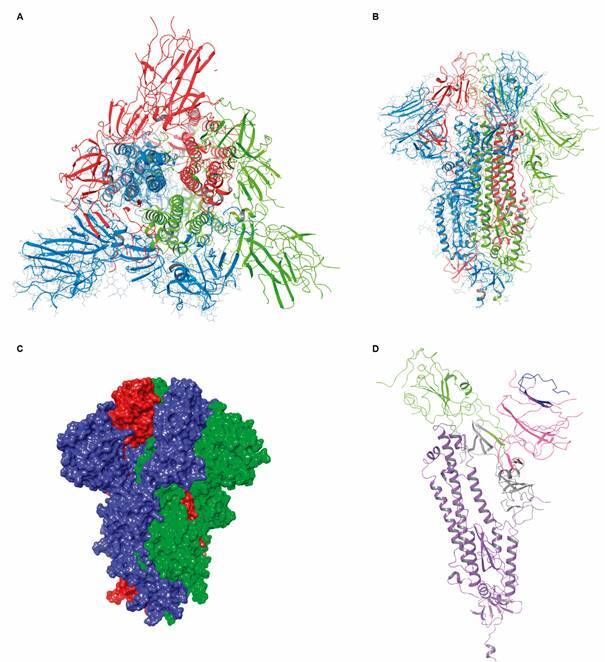
tertiary and quaternary structure of extracellular domain of severe acute respiratory syndrome coronavirus 2 (SARS-CoV-2) S protein (PDB: 6VXX, 2.8 Å). (A) Top view of the protein trimer. Each monomer is distinctly coloured (blue, light green and red). (B) Side view of the protein. (C) VdW surface of each monomer. (D) Subunits S1 and S2 (violet) of extracellular domain. The S1 subunit is further divided into N terminal (NTD, pink) and C-terminal domains (CTD, green). At the tip of the NTD, the ganglioside-binding domain (GBD) is coloured in blue. CTD is also the receptor-binding domain (RBD).

CoVs S proteins require proteolytic priming or cleavage to become fusion competent.^59^ This is achieved by host proteases (e.g., TMPRSS2, cathepsin L, furins) which cleave the S2 subunit of S protein at two main positions: S1/S2 interface and S2’. The latter is located immediately upstream of the fusion peptide (FP), the fusion functional element of S2. Contrary to SARS-CoV, SARS-CoV-2 S2 subunit also has an additional cleavage site for furin proteases in the S1/S2 region, something that also occurs in MERS-CoV.^60^ It is still unclear its role in SARS-CoV-2 infection, but it is speculated that the ubiquitous expression of furins may increase cell and tissue tropism of SARS-CoV-2 in comparison to SARS-CoV, as well altering its transmissibility and pathogenicity.^60^ ^,^ ^117^ Taken together, host proteases represent potential targets for anti-SARS-CoV-2 drugs, and thus some of them will be discussed in the following topics.^60^ ^,^ ^64^ ^,^ ^119^

CoVs E proteins are small integral membrane polypeptides (76 - 109 aa) encoded by subgenomic RNAs. In SARS-CoV, they are found in virions, but also in large amounts in the ERGIC where they participate in virus budding and trafficking. Their structures are divided into three main domains: N-terminal, transmembrane (TMD) and C-terminal domain. TMD is able to form pentameric α-helical bundles creating ion conductive pores in membranes.^120^ ^,^ ^121^ ^,^ ^122^ The ion channel (IC) activity of E protein has been proposed to alter ion homeostasis, as well induce inflammatory response, which may lead to pulmonary damage.^123^ ^,^ ^124^ Thus, the use of inhibitors of IC activity may represent a possible therapeutic strategy for CoVs-related diseases, including COVID-19.^67^ ^,^ ^122^ ^,^ ^123^ ^,^ ^125^ This can be exemplified by the FDA-approved drugs gliclazide (17), a sulfonylurea administered to non-insulin-dependent diabetes mellitus patients, and memantine (18), an N-methyl-D-aspartate receptor antagonist used in the management of Alzheimer’s disease, which have shown IC inhibitory activity in bacteria expressing SARS-CoV-2 E proteins.^126^

*Host cell targets* - A key step in SARS-CoVs infection is the attachment of S protein to host angiotensin-converting enzyme 2 (ACE2), a membrane-bound carboxypeptidase (family: M2, clan: MA).^25^ ^,^ ^65^ ^,^ ^69^ This enzyme catalyses the hydrolysis of angiotensin I and angiotensin II into angiotensin-(1-9) and angiotensin-(1-7) peptides, respectively.^127^ ACE2 is expressed in the upper respiratory system, type I and II alveolar epithelial cells in the lungs, heart, endothelial cells, kidney tubular epithelium and other tissues.^128^ Its role as a functional receptor of SARS-CoV-2 S protein in host cells makes this protein a potential drug target to treat COVID-19. There are several therapeutic strategies targeting ACE2 which include: vaccines based on S protein, exogenous administration of a soluble form of ACE2, and administration of small molecules [e.g., arbidol (19), an anti-influenza drug] or antibodies (e.g., STI-1499, an anti-spike antibody) to block the interaction of ACE2 with S protein.^66^ ^,^ ^129^ ^,^ ^130^ Additionally, compounds could also have an anti-SARS-CoVs activity by disturbing ACE2 glycosylation, as proposed for chloroquine (20).^131^

Renin-angiotensin-aldosterone system (RAAS) inhibitors, such as ACE inhibitors [ACE-i, e.g., captopril (21)] and angiotensin receptor blockers [ARB, e.g., losartan (22)], are commonly used in clinics to treat hypertension and cardiovascular/renal diseases.^132^ ^,^ ^133^ Recently, some researchers have debated over the administration of these drugs to patients with known or suspected COVID-19.^133^ ^,^ ^134^ ^,^ ^135^ ^,^ ^136^ The main reason for this discussion is that RAAS inhibitors may induce ACE2 expression, which, in theory, could increase the severity of the infection.^133^ ^,^ ^136^ Nonetheless, evidence suggests that these drugs may protect patients from lung injury by suppressing angiotensin II signaling mediated by angiotensin receptor 1 (AT1R).^135^ ^,^ ^136^ ^,^ ^137^ Further investigation is still required to determine whether ACE-i and ARB have a beneficial or deleterious role in COVID-19 treatment.^134^

In order to become fusion-competent, SAR-CoVs S proteins must be cleaved by host proteases. In SARS-CoV-2, this process appears to be mediated primarily by TMPRSS2 in the plasma membrane.^65^ TMPRSS2 (transmembrane protease, serine 2) is a transmembrane protein (predicted to have 492 aa) from the serine protease class (family: S1 clan: PA).^69^ ^,^ ^138^ It is highly expressed in the epithelial cells of the prostate, and relatively less in lungs, colon, liver, kidney, and pancreas. Its physiological function is still unclear.^138^ TMPRSS2 has a major role in SARS-CoV-2 cell entry and replication, and thus represents an interesting therapeutic target since its inhibitors could potentially block virus infection in its initial stages.^65^ ^,^ ^138^ Potential TMPRSS2 blockers include some serine protease inhibitors [e.g., camostat mesylate (23)], commercially available compounds [e. g., ZINC64606047 (24), 3-[[5-(3-fluorophenyl)pyrimidin-2-yl]amino~(N)-(4-methyl phenyl)benzamide]] and natural products [e.g., withanone (25)].^65^ ^,^ ^139^ ^,^ ^140^ ^,^ ^141^

In the absence of exogenous or membrane-bound proteases (e g. TMPRSS2) to promote SARS-CoVs infection on cells’ surface, the virus can also be internalised via endocytosis, also known as “late pathway”.^59^ This process comprises several factors that operate in a sequential and partially overlapping fashion.^142^ Endocytosis initiates with the recruitment of endocytic coat proteins (e. g., clathrins) from the cytosol to gather on the inner leaflet of the plasma membrane. Then, protein-coated pits bud into vesicles originated from plasma membranes and fuse with each other or with pre-existing early endosomes.^142^ ^,^ ^143^ Gradually, early endosomes mature to late endosomes which have an acid environment (approx. pH 5.5), activating endosomal cathepsin L cysteine proteases (family: C1, clan: CA).^59^ ^,^ ^69^ ^,^ ^143^ These enzymes are responsible for triggering the fusion activity of S protein and subsequent insertion of viral SARS-CoV gRNA into the cytosol, especially in cells with lower expression of TMPRSS2.^64^ ^,^ ^65^ Some key elements of endocytosis have been explored as potential targets for anti-SARS-CoV drugs, such as agents that increase endosomal pH (avoiding cathepsin L activation) [e.g., chloroquine (20) and hydroxychloroquine (26)] and cathepsin L inhibitors [e.g., teicoplanin (27), a glycopeptide antibiotic].^49^ ^,^ ^131^ ^,^ ^144^

Cytokines are short-lived small size proteins (15-20 kDa) that exert an important role in autocrine, paracrine, and endocrine signaling controlling the development and function of several immune and nonimmune cells.^145^ ^,^ ^146^ They are classified in families based on certain properties of their receptor complexes, such as their structure, specificity and composition.^145^ One example is Interleukin (IL)-6 family which includes several cytokines, such as IL-6, that use the common signaling receptor subunit glycoprotein 130 kDa (gp130). IL-6 signaling requires the binding of IL-6 to IL-6 receptor (IL-6R) which consists of a soluble IL-6 binding domain (CD126) and membrane-bound gp130. IL-6 acts as the main inducer of fever, inflammation and of hepatic acute phase proteins. Therefore, IL-6/IL-6R antagonists have a therapeutic effect in inflammatory diseases.^145^ ^,^ ^146^ Their use in COVID-19 treatment may be beneficial to avoid the “cytokines storm” syndrome that some patients with the severe form of the disease may develop.^147^ This deleterious event is caused by an amplified immune response and cytokine release that can damage the organs, including the lungs.^66^ Tocilizumab and sarilumab are two examples of humanised monoclonal antibodies that act as IL-6R antagonists which are currently under clinical trials for COVID-19 treatment.^148^

*Other targets* - Apart from SARS-CoVs infection, some relevant molecular targets of other viral diseases and host metabolism have also been investigated in COVID-19 drug discovery, such as viral neuraminidases and the DPP4 cell receptor. Neuraminidases (NA) or sialidases, are glycoside hydrolases (family: GH34) largely found attached to the envelope of influenza viruses.^149^ ^,^ ^150^ They catalyse the hydrolysis of glycosidic bonds between sialic acid and adjacent sugar residues (EC 3.2.1.18) in glycoproteins, glycolipids and oligosaccharides.^149^ NA is mainly responsible for cleaving sialic acid acids from cell receptors and on carbohydrate side chains of nascent virion, facilitating its release from infected cells.^150^ Its critical role in virus infection and proliferation has been exploited by NA inhibitors [e.g., oseltamivir (28)] which are administered in clinics to combat influenza infections. Apparently, this therapeutic strategy was employed at the beginning of COVID-19 outbreak during the peak of influenza season in China when the etiologic agent of this disease was yet unknown. So far, there is no evidence that NA inhibitors may have a role in COVID-19 management.^66^

Dipeptidyl peptidase-4 (DDP4), also known as adenosine deaminase complexing protein 2 or T-cell activation antigen CD26, is a type II transmembrane glycoprotein of 766 aa largely expressed in many tissues (e.g. lungs and immune cells).^151^ ^,^ ^152^ DDP4 belongs to serine protease group (family: S9, clan: SC) and catalyses the hydrolysis of N-terminal dipeptide, Xaa-Yaa-|-Zaa-, (preferentially when Yaa is a proline, as long as Zaa is neither proline or hydroxyproline), from a variety of peptide substrates (EC 3.4.14.5), such as chemokines, neuropeptides, vasoactive peptides and growth factors.^69^ ^,^ ^71^ ^,^ ^152^ Therefore, it participates in many physiologic and pathological processes, including glucose/insulin metabolism and immune/inflammatory response.^153^ In addition, DPP4 acts as the functional cell receptor of MERS-CoV S protein, helping virus entry into the cell. Theoretically, this role may also be played in SARS-CoV-2 infection, as predicted by computational analysis.^154^ Recently, there has been a discussion about the use of DDP4 inhibitors, such as sitagliptin (29) (anti-diabetic drug), in the treatment of COVID-19.^152^ ^,^ ^153^ ^,^ ^155^ ^,^ ^156^ In principle, these inhibitors could be beneficial for type 2 diabetes patients (or even without diabetes) with COVID-19 since it could potentially block virus cell entry/replication, as well suppress inflammatory response.^152^ ^,^ ^155^ However, there is not enough evidence yet to prove this hypothesis.^152^ ^,^ ^153^ ^,^ ^156^

Repositioning studies in progress for the treatment of COVID-19

*Computational approaches* - The SARS-CoV-2 pandemic is creating a fertile ground for computational approaches to identify viable therapeutic options in the short-term, with the number of published studies growing rapidly.^157^ ^,^ ^158^ Classical target- and ligand-based strategies (e.g., molecular docking and similarity analysis), are being applied to FDA-approved drugs and compounds in clinical trials to explore possible interactions with viral proteins.^75^ ^,^ ^159^ ^,^ ^160^ Artificial intelligence (AI) methods are also being extensively applied to large molecule databases to find existing drugs that could be repurposed based on previously reported antiviral activities.^103^ ^,^ ^161^ ^,^ ^162^ ^,^ ^163^ AI approaches can even explore hidden connections between drugs, human and viral targets to find novel bioactivities and even combinations of drugs.^52^ ^,^ ^161^ ^,^ ^164^ These studies emphasize how important computational methods are in modern drug discovery to analyse the huge amount of biomedical data available. Table I summarises a selection of studies that suggested potential drugs for repurposing. Below we will describe the main computational methods and results. Smith and Smith performed a large virtual high-throughput screening to identify drugs, natural products and metabolites that could disrupt the interaction between SARS-CoV-2 S protein and human ACE2 receptor. The authors used a supercomputer called SUMMIT to conduct the simulations, which included structural modeling of the S protein:ACE2 complex, generation of an ensemble of conformations for the complex and molecular docking.^160^ The ensemble generation was a crucial step in this work, allowing the authors to explore different conformations of the complex and identify drugs that could interact with binding sites not easily identified in static conformations.

**TABLE I:** *In silico* studies of drug repurposing for coronavirus disease 2019 (COVID-19)

Repurposed drug	Previous use	Predicted targets	Discovery origin	Experimental evidence	Reference
Pemirolast (30), nitrofurantoin (31), isoniazid pyruvate (32), eriodictyol (33), cepharanthine (34), ergoloid (35) and hypericin (36)	Multiple	Disrupting spike protein (S-protein) and ACE2 receptor interaction or inhibit S-protein and block ACE2 receptor	Ensemble docking	Natural products, such as eriodictyol (33), cepharanthin (34), ergoloid (35) and hypericin (36) have shown antiviral activity against coronaviruses, including severe acute respiratory syndrome coronavirus (SARS-CoV).^*a*^	^160^
Mefuparib hydrochloride (CVL218) (37)	Cancer	Nucleocapsid (N protein)	Knowledge-graph docking *in vitro* and *in vivo* assays	Mefuparib (37) achieved an EC_50_ of 5.12 μM.	^164^
Atazanavir (12) and efavirenz (38)	HIV	SARS-CoV-2 3C-like protease (M^pro^)	Molecule transformer drug-target interaction (MT-DTI)	Atazanavir (12) can been found in lungs after administration and have shown inhibitory activity against M^pro^.^*b*^	^103^
Baricitinib (39)	Rheumatoid arthritis	Human AP2-associated protein kinase 1 (AAK1)	Knowledge-graph	A pilot trial indicated that baricitinib (39) improved clinical parameters (e.g., cough and dyspnea) in a small group of patients.^*c*^	^161^
Toremifene (40), sirolimus (41), mercaptopurine (42), irbesartan (43)	Multiple	Multiple targets in humans and coronaviruses	Systems pharmacology-based network and human protein-protein (PPI) network	Antiviral activities against coronaviruses has been described for selective estrogen modulators toremifene (40), immunosuppressants sirolimus (41) and mercaptopurine (42) and angiotensin receptor blockers irbesartan (43).^*d*^	^52^

*a*: Cepharanthin (34) has been shown to decrease the expression levels of S-protein in MRC-5 cells infected with *Human coronavirus OC43* (*HCoV*-*OC43*).^165^ Flavonoids have been described as inhibitors of M^pro^ and ligands of the S-protein of SARS-CoV, which could give insights on possible antiviral activities of eriodictyol (33), hypericin (36) and other flavonoids on SARS-CoV-2.^166^ ^,^ ^167^ *b*: a recent study showed that Atazanavir (12) inhibited M^pro^ *in vitro* with greater potency than lopinavir (1).^168^ *c*: despite the small size (12 patients) and open label, non-randomised format, baricitinib (39) showed potential safety when used in combination with lopinavir (1) / ritonavir (2).^169^ As of August 3rd 13th, 2020, there are three ongoing clinical trials with baricitinib (39): NCT04421027 (phase 2, randomised and double blind), NCT04320277 (phases 2 and 3, non-randomised and open-label) and NCT04321993 (phase 2, non-randomised and open-label). *d*: the authors described previous antiviral activities for the most promising drugs, including against other coronaviruses (e. g., MERS-CoV and SARS-CoV), showing a correlation between the original target of each drug and coronaviruses target.

Using the GROMACS molecular dynamics simulation suite, six different clusters of conformations were generated by restrained temperature replica-exchange molecular dynamics simulation (T-REMD) and submitted to docking with Autodock Vina using the SWEETLEAD compound collection.^170^ ^,^ ^171^ ^,^ ^172^ The authors explored two strategies, consisting of disrupting the S protein: ACE2 complex and preventing its formation by docking on the isolated proteins. From this analysis, four compounds [pemirolast (30), nitrofurantoin (31), isoniazid pyruvate (32) and eriodictyol (33)] displayed potential to disrupt the S protein: ACE2 interaction, and three natural compounds [cepharanthine (34), ergoloid (35) and hypericin (36)] showed potential to block host recognition.^160^ Chemical structures for compounds 30-49 are shown in Fig. 12.

**Fig. 12:**
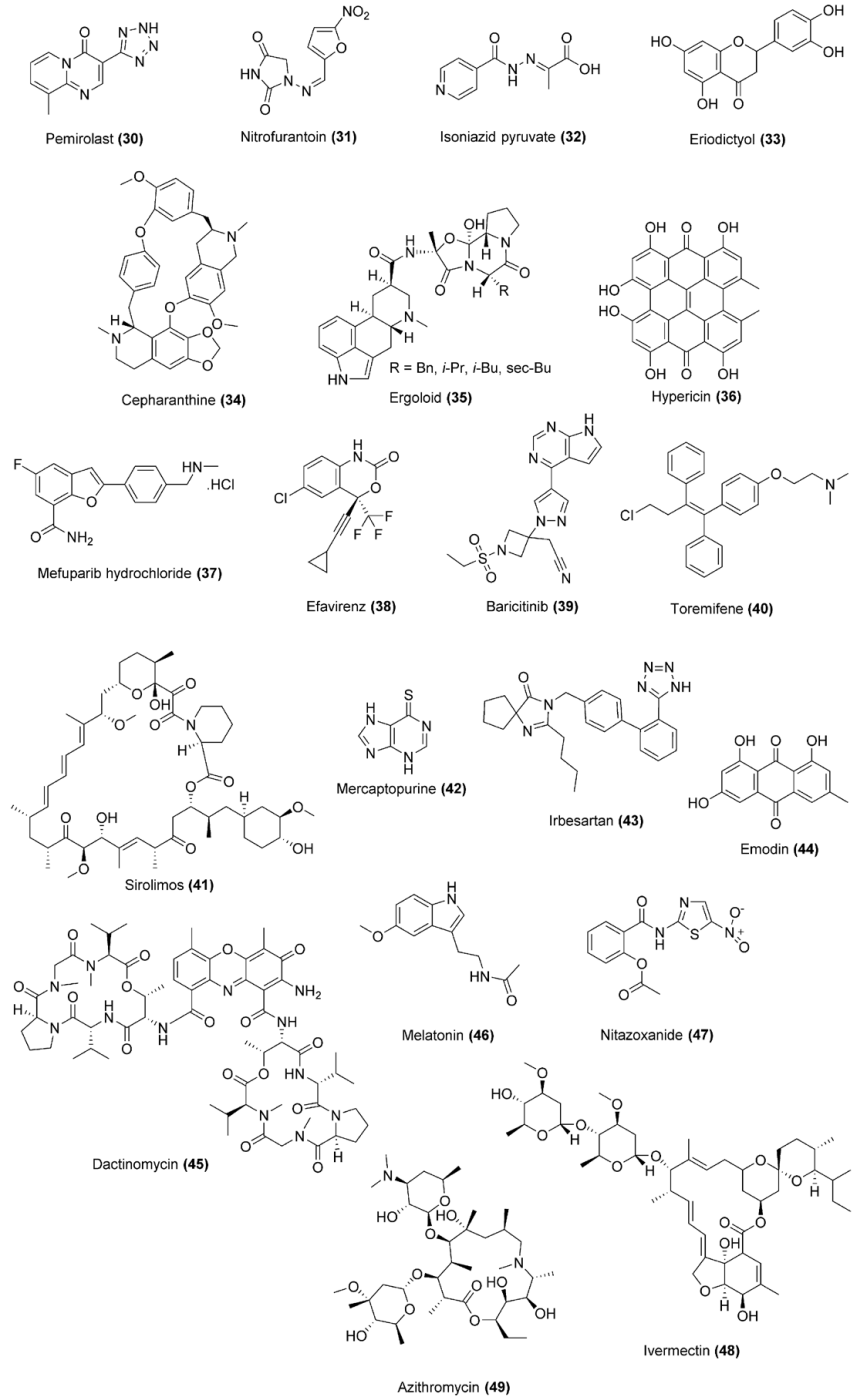
chemical structures for drug repurposing candidates and compounds 30-49 under investigation against coronavirus disease 2019 (COVID-19).

Ge and coworkers constructed a virus-related knowledge graph (or network) by combining seven networks with information about target-drug and protein-protein interactions, molecule similarity and sequence similarity of human and viral sources to predict drugs targeting SARS-CoV-2.^164^ Well known biological and interaction databases were employed, such as DrugBank, ChEMBL,⁠ BindingDB,⁠ and UniProt⁠.^173^ ^,^ ^174^ ^,^ ^175^ ^,^ ^176^ The final knowledge graph was assembled by merging the nodes and edges of the seven networks, where each node represent drugs or targets, while the edges between them are the identified relations, such as similarity (i.e., molecular and sequence similarities) and drug-target or target-target interactions.^164^⁠ A graph convolutional network (GCN) was used to learn and extract the hidden information on the nodes and edges of the knowledge-graph, allowing the authors to access novel drug-target and target-target interactions and find molecules that could be repurposed to SARS-CoV-2.^164^ GCN’s are powerful neural networks that can access the rich information within the nodes of a graph and return insights about possible relations (e.g., potential drugs and targets interactions), representing a valuable tool in the modern drug discovery scenario, where massive biological data are available in databases such as DrugBank and ChEMBL.^177^ ^,^ ^178^

The final knowledge-graph was then mined to gather an initial list of drugs, which was further refined by extracting previous evidence of antiviral activity from PubMed using a deep learning method called Biomedical Entity Relation Extraction (BERE) and manual inspection.^179^ In a subsequent step, the authors elaborated a final list of drug candidates using transcriptome analysis of ten SARS-CoV patients. The poly-ADP-ribose polymerase 1 (PARP1) inhibitor, mefuparib hydrochloride (CVL218) (37), currently in Phase I clinical trials, was identified as a potential drug for repurposing. *In vitro* and *in vivo* validation showed promising inhibition and safety profiles. Concretely, compared to arbidol (19), one of the standard treatments for COVID-19 in China, CVL218 showed higher antiviral efficacy and higher concentration in lungs of rats.^164^ The authors also found that CVL218 significantly inhibited the production of IL-6 induced by the CpG oligodeoxynucleotide 1826 (CPG-ODN1826) in peripheral blood mononuclear cells (PMBC) indicating it might be an alternative to treat the inflammation caused by SARS-CoV-2. Furthermore, CVL218 showed favorable pharmacokinetics and toxicity profiles in rats and monkey models.^164^⁠ This integration of artificial intelligence with wet-lab experiments highlights the importance of *in silico* methods to mine large amounts of data from databases and accelerate the discovery of potential drugs for the CoVid-19 pandemic.

Beck et al. used a natural language processing (NLP)-based approach to estimate the binding affinity of 3,410 FDA-approved drugs against potential targets of SARS-CoV-2, including 3CL^pro^, S protein, RdRP, helicase, endoRNAse, 3’-to-5’- exonuclease and 2’-O-ribose methyltransferase.^103^ The premise of their approach is analogous to understanding text in different languages, for instance by learning the semantic relationships of words to execute a task, such as predicting the most probable word in a text or the sentiment expressed by it.^180^ ^,^ ^181^ ^,^ ^182^⁠ Their model, called molecular transformer-drug target interaction (MT-DTI) was trained on 1 billion SMILES strings and the FASTA sequence of target proteins, which bypass the need for 3D structures (e.g., X-ray) of protein-target complexes.^183^⁠ The pre-training approach allows the model to be used for other related tasks without the need to train from scratch, which is especially important when not enough data is available, which is the case for SARS-CoV-2 inhibitors. In addition, this approach is able to transfer more general features learned by the model, making it extremely flexible to deal with related tasks.^184^ ^,^ ^185^

Therefore, the task consisted of training the model to understand the chemical structure of small compounds and protein targets. The authors found that atazanavir (12), remdesivir (8) and efavirenz (38) are potential inhibitors of M^pro^, while atazanavir (12) also yielded nanomolar predicted binding affinity for RdRP, helicase, endoRNAse, 3’-to-5’- exonuclease and 2’-O-ribose methyltransferase.^103^⁠⁠ It is important to highlight that although these drugs were predicted as nanomolar inhibitors, experimental confirmation is essential to validate the computational analysis.

Researchers at the UK-based company BenevolentAI (https://www.benevolent.com/) analysed medical data from different sources to create a knowledge graph of biomedical information, showing possible drug-target interactions on its nodes and edges (similar to the previously mentioned approach adopted by Ge et al.) to explore new strategies for SARS-CoV-2.^161^ ^,^ ^164^ Among the drugs identified by mining the hidden information within the graph, there were 378 inhibitors of the P2-associated protein kinase 1 (AAK1), a regulator of viral endocytosis in AT2 alveolar epithelial cells.^161^ Inhibitors of AAK1 could then potentially block viral entry into alveolar cells. However, the authors argued that only one of these drugs, baricitinib (39), a janus kinase inhibitor, has an acceptable safety profile. In addition, the low therapeutic dosing of baricitinib (39) (2mg or 4mg daily) makes it a promising drug for repurposing.^161^ Baricitinib (39) will be further discussed below.

Zhou et al. developed a network-based approach to identify potential repurposable drugs based on the interactions between their human targets and coronavirus-associated proteins.^52^ The premise is that protein targets that are associated with viral infections are part of a subnetwork of the human protein-protein interaction network. The targets of a repurposable drug should be in or close in proximity to this subnetwork. The authors identified many potential targets, such as Poly [ADP-ribose] polymerase 1 (PARP-1), Glycogen synthase kinase 3 (GSK-3), and also biological pathways that could be explored, such as NF-kappa B signaling.^52^⁠ The most interesting drugs selected by the authors are from a diverse set, including toremifene (40) (selective estrogen receptor modulator), sirolimus (41) (immunosuppressant), mercaptopurine (42) (antineoplastic) and irbesartan (43) (antihypertensive). An interesting finding was that the selected molecules and targets have reported links to viral infection and some have been used for treatment of Coronavirus-related disease, such as emodin (44) in SARS-CoV, and mercaptopurine (42) and toremifene (40) in SARS-CoV and MERS-CoV.^52^ In addition to identifying repurposable drugs, possible synergistic combinations between them were also suggested by the network, such as sirolimus (41) and dactinomycin (45), and mercaptopurine (42) and melatonin (46).^52^

*In vitro and in vivo studies* - Genome and replication kinetics of SARS-CoV, MERS-CoV and SARS-CoV-2 viruses are very similar, suggesting that these diseases could be treated by a single drug that modulates the activity of a common target or mechanism among them.^52^ ^,^ ^186^ So far, no clinically available antiviral drugs have been developed for any of these three viruses.^187^ This fact demonstrates that we did not learn a suitable strategy for treatment from these two prior diseases, and we are not yet prepared to deal with this new coronavirus epidemic, regarding therapeutic options.^188^

Experimental target validation (e.g., by genetic and proteomic approaches), cellular *in vitro* and *in vivo* animal models of SARS-CoV-2 infection, are pivotal for the discovery of molecular pathways involved in pathogenesis, supporting the discovery of new drugs.^189^ ^,^ ^190^ SARS-CoV-2 genome encodes less than 30 proteins that can be explored for generating new avenues for further research. Heterologous viral protein expression could be used in a myriad of structural and functional target-based biochemical studies.^191^ These assays do not need to be performed in biosafety level 3 laboratory, democratising the early research on searching for a new drug candidate for COVID-19 treatment.^192^ Genome-wide expression studies, nucleotide sequences, protein structures and associated sequences are available online providing a foundation for preclinical drug discovery and development research against SARS-CoV-2 (Available from: https://www.ncbi.nlm.nih.gov/genbank/sars-cov-2-seqs/). It is good to point out that many drugs show *in vitro* effect (e.g., target and/or cellular assays) that may not last *in vivo*, in animal models, including humans. An ideal animal model of COVID-19 should reflect the clinical signs, viral replication and pathology reported in humans.^193^ *In vivo* animal infection experiments with SARS-CoV-2 require infrastructure of a biosafety level 3 laboratory, restricting scientific research to well-equipped research groups. Animal models are indispensable, because they could identify toxic hits or molecules that could enhance COVID-19 pathogenicity. Table II lists some compounds that have the potential to be clinically tested and that will be discussed in more details below in this topic.

**TABLE II:** *In vitro* and *in vivo* studies of drug repurposing for coronavirus disease 2019 (COVID-19)

Repurposed drug	Previous use	Targets	*In vitro* potency (EC_50_)	Selectivity index	Reference
Remdesivir (8)	Ebola	RNA polymerase	0.77 μM	129.9	^194^
Chloroquine (20) / Hydroxychloroquine (26)	Malaria / Lupus and rheumatoid arthritis	Acidification in endosomes and lysosomes, glycosylation of cellular virus receptors and modulation host immune response	1.13 μM / 0.72 μM	37.1 to 100.8 / 14.4 to 61.5 depending on multiplicities of infection	^194^ ^,^ ^195^ ^,^ ^196^
Ritonavir (2) + lopinavir (1)	HIV	3-chymotrypsin-like protease (3CL^pro^) and papain-like protease (PL^pro^)	6.6 to 17.1 μM for Lopinavir (1)	ND	^197^
Nitazoxanide (47)	Broad-spectrum antiparasitic and antiviral	Still needs confirmation	2.1 μM	16.8	^194^
Ivermectin (48)	Broad-spectrum antiparasitic	Still needs confirmation	2 μM	ND	^198^
Teicoplanin (27)	Antibiotic for gram-positive bacteria	Still needs confirmation	1.7 μM	ND	^199^

Remdesivir (8), a nucleotide analog pro-drug developed by Gilead Sciences Inc. to fight Ebola, acts on viral replication by inhibiting RNA polymerase.^96^ This molecule was also active against SARS and MERS viruses.^200^ ^,^ ^201^ ^,^ ^202^ Wang et al. demonstrated reduced viral copy numbers in cell culture supernatant of Vero E6 cells infected with nCoV- 2019BetaCoV/Wuhan/WIV04/20192, in the presence of varying concentrations of Remdesivir (8).^194^ This compound blocked virus infection at low-micromolar concentration (EC_50_ = 0.77 μM) and showed a high selectivity index (> 100). The authors also disclosed that remdesivir (8) inhibited virus infection efficiently in human liver cancer Huh-7 cells.

Chloroquine (CQ; 20) and its less toxic derivative, hydroxychloroquine (HCQ; 26), are used to treat malaria and lupus/rheumatoid arthritis, respectively. Both drugs have already been described as possible antivirals.^195^ ^,^ ^203^ ^,^ ^204^ CQ (20) and derivatives probably act by decreasing acidity in endosomes and lysosomes, intervening on glycosylation of cellular virus receptors and modulating host immunological activity.^118^ ^,^ ^205^ Studies in cultures of Vero E6 cells have suggested that HCQ (26) can affect the virus SARS-CoV-2 in both entry and post entry stages at host cells.^194^ This molecule presented an EC_50_ value of 1.13 μM and selectivity index of 88.5. HCQ (26) is also being studied *in vitro* as a SARS-CoV-2 infection inhibitor acting on both virus entry, as well as post-entry steps on host cells.^206^ Comparing dose-response curves of the two compounds against an *in vitro* model of SARS-CoV-2 infection on Vero E6 cells, demonstrated that CQ (20) is slightly more potent than HCQ (26) at four different multiplicities of infection (0.01, 0.02, 0.2 and 0.8): EC_50_ 2.71, 3.81, 7.14 and 7.36 μM for CQ versus 4.51, 4.06, 17.31, and 12.96 μM for HCQ, respectively. RNA copy numbers in the cell supernatant at 48 h post infection were measured. The authors also measured the cytotoxicity of both compounds at the same *in vitro* model and demonstrated that the 50% cytotoxic concentration (CC_50_) values of CQ (20) and its derivative were remarkably similar ~ 250 μM. In another study Yao et al. demonstrated that HCQ (26) was more potent than CQ (20) to inhibit *in vitro* SARS-CoV-2 infection (EC_50_ 0.72 vs 5.47 μM).^196^ The authors also performed *in vitro* physiologically based pharmacokinetic models for both drugs, separately. Interestingly, when comparing toxicity in five animal models, McChesney et al. demonstrated that HCQ (26) is two to three times less toxic than CQ (20) itself.^207^ It is also good to point out that chloroquine (20) has shown *in vitro* activity against many different viruses, but no significant beneficial effect on animal models.^208^

The association ritonavir (2)/lopinavir (1) is used together with other antiretrovirals for the treatment of human immunodeficiency virus since the beginning of the century.^209^ Ritonavir (2) is a potent CYP3A inhibitor therefore inhibiting the metabolism of lopinavir (1), increasing its plasma levels. Both are reported as peptidomimetic molecules that inhibit HIV-1 protease activity in a competitive manner.^210^ The M^pro^ plays a major role in homeostasis of viral polyproteins essential for viral function and replication, being considered a validated drug target.^211^ This combination may be useful against SARS-CoV-2 virus by acting on its main protease, an enzyme essential in processing polyproteins translated from viral RNA.^212^ Choy et al. demonstrated that it inhibits SARS-CoV-2 replication in Vero E6 cells with EC_50_ value of 26.6 μM.^197^ These results corroborate with the study of de Wilde et al. that demonstrated antiviral *in vitro* effect of lopinavir (1), but not ritonavir (2), against SARS-CoV, MERS-CoV, and hCoV-229E, with mean EC_50_ varying from 6.6 to 17.1 μM.^213^

Other compounds were also tested against SARS-CoV-2 *in vitro*. Nitazoxanide (47), a broad-spectrum antiparasitic and antiviral, inhibits a low-micromolar range with an EC_50_ of 2.1 μM but has a selective index of only 16.8.^194^ Ivermectin (48), another broad spectrum antiparasitic agent also demonstrated *in vitro* anti-SARS-CoV-2 activity. Its EC_50_ was determined to be 2 μM when added to Vero-hSLAM cells 2 h post-infection and measuring viral RNA at 48h post-infection.^198^ Teicoplanin (27), an antibiotic used against gram-positive bacterial infections, was found to be active *in vitro* against SARS-CoV-2 with an EC_50_ value of 1.7 μM, but *in vivo* efficacy still needs to be determined.^199^ A robust preclinical drug discovery pipeline comprising *in vitro,* and *in vivo* models of SARS-CoV-2 infection is particularly important to identify new antivirals for human COVID-19 treatment. This pipeline can munition clinical studies with molecules that have a higher chance to become a drug, decreasing attrition rates.

*Clinical studies* - Over 1452 clinical trials have been unveiled, until the publication of this manuscript, focusing on COVID-19 treatment interventions (Available from: https://clinicaltrials.gov/ct2/results?cond=COVID-19). With a growing number of patients suffering from acute severe respiratory symptoms and hospital capacities reaching its limit, therapeutic options are urgently essential to avoid human mass deaths. Drug repurposing approaches of approved (with safe and effectiveness proven) and unapproved molecules, that were promising in pre-clinical and early stages of clinical studies of SARS and MERS, are in vogue. With this premise, World Health Organization (WHO) organised a simplified dynamic platform comparing the effectiveness of treatment strategies around the globe. This clinical trial design, called SOLIDARITY, can shrink by 80% the time of clinical studies, compared with the “gold standard” double-blind, placebo-controlled trials. This strategy could overcome the uncertainty of multiple small trials that do not produce a solid base necessary to establish the relative success of arising probable treatments. On the other hand, this shorter time required reflects the compassionate characteristic of the studies, that cannot rule out placebo effects and patient severe adverse effects, including death.^214^ Currently, WHO is focusing on four most promising therapies: remdesivir (8); CQ (20) or HCQ (26); ritonavir (2)/lopinavir (1); ritonavir (2) /lopinavir (1) plus interferon-beta, an immune response modulator.^44^ A selection of studies with these drugs, alone or in combination, are summarised in Table III. For other studies, the reader can refer to Kupferschmidt and Cohen, which summarised several clinical studies on drug repurposing for Covid-19 reported so far.^44^ It is worth noting that many clinical studies are beginning to be carried out in different parts of the world and their number is increasing rapidly day by day.

**TABLE III:** Clinical studies of drug repurposing for coronavirus disease 2019 (COVID-19)

Repurposed drug	Study characteristics	Estimated enrolment	Dosage	Characteristics of the study and conclusion	Clinical trial and recruitment status
Remdesivir (8)	Phase III, double-blind, randomised, placebo-controlled, multicentre	308 patients	200 mg Intravenously on the first day plus 100 mg once daily, for more nine consecutive days	Adult patients with mild and moderate COVID-19, no published results	NCT04252664 (suspended by epidemic control in China with no eligible patients)
		237 patients		Adult patients with severe COVID-19, remdesivir (8) was not associated with clinical benefits in severe forms. However, an observed numerical reduction in time to clinical improvement in patients treated earlier, but still requires confirmation	^215^; NCT04257656 (terminated by epidemic control in China with no eligible patients)
		1063 patients		Remdesivir (8) treated patients with lower respiratory tract infection have a shorter time to recovery compared to placebo group (11 vs 15 days)	^216^; NCT04280705
Chloroquine (20)	Multicentre	> 100 patients	500 mg per day, for 10 days	Compilation of various clinical studies that are in course, authors conclude that the dosage used could be sufficient	^217^
	Phase IIb Double-blind, randomised	81 patients	600 mg twice daily for 10 days and 450 mg twice on the first day, once daily for more 4 days	Higher dosage of chloroquine (20) has toxic effects and increased lethality, with any clinical benefit	^218^
Hydroxychloroquine (26)	non-randomised, non-double-blind, non-placebo-controlled	36 patients	600 mg of hydroxychloroquine (26) daily for 10 days	Hydroxychloroquine (26) significantly reduces viral load despite small sample size	^219^
Ritonavir (2)/lopinavir (1)	Randomised, controlled, open-label	199 patients	400 mg/100 mg twice a day for 14 days	No benefit was observed with lopinavir (1)-ritonavir (2) treatment compared to standard care in severe Covid-19 patients	^220^
Ritonavir (2)/lopinavir (1) plus interferon	Phase II, randomised, controlled, open-label	127 participants	400 mg/100 mg twice a day for 14 days; Interferon Beta-1B plus 0.25 mg subcutaneous injection alternate day for three days	Completed with no published results until this moment	NCT04276688

Remdesivir (8), a nucleoside analogue that acts by inhibiting RNA synthesis, is already in clinical studies for treatment of COVID-19.^96^ This prodrug was already investigated in clinical trial testing for Ebola virus with no effect but showed efficiency against different types of coronaviruses in cell culture and animal models.^200^ ^,^ ^221^ From the drugs in the SOLIDARITY trial, remdesivir (8) has the best potential to be used in clinics, having a low toxicity profile to humans.^44^ This molecule was used compassionately in the first COVID-19 patient diagnosed in the United States by intravenous treatment and improved patient clinical condition.^222^ Grein et al. reported a study in a cohort of patients hospitalised for severe COVID-19 who were treated with compassionate-use remdesivir (8), clinical improvement was observed in 68% of the patients (36 of 53).^223^ There are two clinical trials at phase III, being designed to evaluate the efficacy and safety of parenteral remdesivir (8) in mild/moderate (NCT04252664) and severe (NCT04257656) hospitalised adults with COVID-19.^224^ These trials are evaluating intravenous remdesivir (8) at a dose of 200 mg on the first day plus 100 mg once daily, for an additional 9 consecutive days. The randomised, double-blind, placebo-controlled, multicentre trials were recently suspended (NCT04252664) or terminated (NCT04257656) because no eligible patients for enrollment were found, due pandemic control in China.^215^ The study NCT04257656 showed that remdesivir (8) was not associated with statistically significant clinical benefits in severe forms of COVID-19. However, the authors observed a numerical reduction in time to clinical improvement in those treated earlier, but still requires confirmation. Another study, performed by Beigel et al. (with the same dosage and treatment period than the previous works cited above) enrolled 1063 patients with lower respiratory tract infection, demonstrated that individuals that were treated with remdesivir (8) had a shorter time to recovery than placebo group (11 days compared with 15 days).^216^ Even with a small scientific ballast, due to limited information about safety and effectiveness of using remdesivir (8), U.S. Food and Drug Administration (FDA) approved its emergency use on severe COVID-19.^225^ The approval was based on review of the topline data on two studies that are recruiting patients, (NCT04280705) and (NCT04292899). Other clinical studies are being conducted to address the effect of remdesivir (8) on COVID-19 treatment (Available from: https://clinicaltrials.gov/ct2/results?cond=COVID-19&term=remdesivir&cntry=&state=&city=&dist=).

Other Phase III clinical trials evaluated for COVID-19 treatment included CQ (20)/HCQ (26). These studies were endorsed by promising *in vitro* data that suggested an impairment of viral replication by acting on endosome-mediated viral entry or later phases of viral replication.^194^ ^,^ ^226^ Both molecules have a well-studied toxicity profile being used for more than half-century as antimalarials, but in some cases can induce heart side effects.^227^ These drugs have already been examined against at least five other viruses with good initial *in vitro* results, but without significant effects in animal models and humans.^205^

Clinical trials to measure the effectiveness of CQ (20) at 500 mg of chloroquine diphosphate (corresponding to 300 mg of chloroquine (20) base) per day, for ten consecutive days on the treatment of COVID-19 were performed.^217^ Based on results for a very limited number of patients (100), it was concluded that CQ (20) presented apparent efficacy and acceptable safety against COVID-19 associated pneumonia. Borba et al. performed a double-blind, randomised clinical trial designed to assess the safety of CQ (20) in two dosages: 600 mg twice daily for 10 days with an initial dose of 450 mg twice daily on the first day, decreasing to once daily for four more days.^218^ The study suggests that the higher dosage should not be recommended for critically ill patients with COVID-19 because of its potential side effects. It is important to note that both doses studied were above the Brazilian recommended dose.

A controversial study performed by Gautret et al. with a small sample size demonstrated that HCQ (26) treatment daily at 600 mg significantly decreases viral load in COVID-19 patients and its effect is reinforced by azithromycin (AZ; 49) combination.^219^ Until this moment there is clinical robustness to support AZ (49) therapy in COVID-19.^228^

Mehra et al. performed a registry analysis of 96,032 hospitalised patients regarding the use of CQ (20) or HCQ (26, combined or not with a macrolide) for treatment of COVID-19.^229^ The observational study verified that with COVID-19 requiring hospitalisation a regimen containing CQ (20) or HCQ (26) had no evidence of benefit, but instead was associated with an increase in the risk of ventricular arrhythmias and a greater hazard for in-hospital death with COVID-19. These findings suggest that these drug regimens should not be used outside of clinical trials and urgent confirmation from randomised clinical trials is needed. After the publication, the paper was retracted, which influenced WHO to return the studies on CQ (20) and HCQ (26).^230^ Recently, on 17 June 2020, WHO stopped the HCQ (26) arm of the Solidarity trials again based on the RECOVERY trials (Available from: https://www.recoverytrial.net/). In this study 1542 patients were randomised to HCQ (26) and compared with 3132 patients randomised to usual care alone. There was no significant difference in any of the parameters evaluated: the primary endpoint of 28-day mortality, hazard ratio and hospital stay duration or other issues. Other study reported that HCQ (26) did not prevent COVID-19 when used as postexposure prophylaxis within 4 days after moderate or high-risk exposure.^231^ Robust data from well-designed control randomised clinical trials with large number of patients is still expected for concluding on the efficacy of hydroxychloroquine (26).

Ritonavir (2) and lopinavir (1) were developed to target HIV-1 protease and postulated to inhibit the 3-chymotrypsin-like protease of SARS and MERS, being associated with improved clinical outcomes.^232^ The combination has beneficial effects in marmoset monkeys infected with the MERS-CoV virus.^233^ (1)/(2) may have clinical efficacy against SARS-CoV-2, as seen in the response against SARS-CoV.^234^ *In vitro* sensitivity and satisfactory response in a preliminary non-randomised clinical trial of SARS-CoV to (1)/(2) have already been reported, encouraging its testing in SARS-CoV-2.^235^ A total of 199 patients confirmed severe SARS-CoV-2 infection were randomly designated to be given either (1)/(2) (400 mg/100 mg) twice a day for 14 consecutive days plus standard care or standard care alone.^220^ This first trial was not promising, no benefit was observed on clinical improvement, mortality and viral loads on severe COVID-19 patients. Other clinical trials are being carried out and should decide whether these drugs are useful for COVID-19 treatment or not.

Ritonavir (2)/lopinavir (1) plus interferon-beta, a cytokine involved in inflammatory modulation, is the last bet of the SOLIDARITY megatrial. Sheahan et al. demonstrated that this combination improves pulmonary function but does not reduce virus replication or severe lung pathology in mice model of MERS-CoV.^236^ This combination also showed promising results in MERS-CoV Infection in a Nonhuman Primate Model.^233^ The study (NCT04276688) administered ritonavir (2)/lopinavir (1) 400 mg + 100 mg/ml twice daily for 14 days and Interferon beta-1b 0.25 mg subcutaneous, every alternate day for 14 days. Another study (NCT04343768) will perform a randomised trial to verify the effects of interferon beta 1a, compared to interferon beta 1b and the base therapeutic treatment in Moderate to Severe COVID-19.

Currently, there is a lot of research around the world examining drugs that can be used for the treatment of COVID-19. Unfortunately, until this moment no therapeutic options are promptly effectively available. Most of the studies have a small number of patients with variable dose and/or duration, fact that hinders a comparison. To successfully undertake the COVID-19 pandemic with new medicines, synchronised clinical trials with randomised, double-blind, placebo-controlled are still needed. Infection prevention by social distancing and supportive medical care, are the only strategies to deal with this disease until this moment.

Patent protection and synthesis of repositionable drugs

The goal of this session is to consider potential obstacles for effective adoption of a repurposed drug to widespread treatment of COVID-19. Both patent protection and scalability of manufacturing processes are key aspects to consider while choosing the best therapeutic option to adopt when dealing with a pandemic.

A patent can be defined as a government license that confers the owner the exclusivity over a new invention or medication. With the patent, the inventor is able to exclude others from making and selling the invention for a determined period of time. When it comes to medication, the market exclusivity granted by the patent generates enormous economic profit for the patent holder, once the company will have the monopoly over the product for around 20 years. Once the patent term finishes, generic companies can start producing and selling the drug, increasing the market competition over the product. A strategy adopted by some companies is to maximise the term of patent of successful products, extending market exclusivity, for instance, increasing economic rewards with the invention. In order to extend patent terms, the companies can apply for new formulations, new routes of administration, new uses of the drug among other strategies.^237^ ^,^ ^238^ This is very important when considering drug repurposing, once those strategies can make it harder to patent a new method of use for the drug, compromising the legal rights of the new repurposed indication. As a consequence, the expected profit associated with repurposing can be severely affected.^39^

Remdesivir (8) a good example of maximising the term of patent is the drug remdesivir (8), which was developed by the American pharmaceutical company Gilead Sciences, Inc. in 2011. Nowadays, (8) has shown promising results as a suitable repurposed drug in the treatment of the COVID-19 disease. Indeed, there are many ongoing clinical trials with this drug in COVID-19 patients.^239^ ^,^ ^240^ Due to the good results concerning remdesivir (8), in January 2020, the Wuhan Institute of Virology applied for a patent on Gilead’s remdesivir (8) for the treatment of coronavirus, in an attempt to protect China’s economic and medical interests. However, remdesivir (8) has already 133 patents related to the coronavirus filed by Gilead Science in 43 countries around the globe since 2011. In fact, the company has a robust patent portfolio that includes structures of remdesivir-related compounds (Family 1), the manufacturing method of the drug (Family 2), the use of remdesivir (8) in treating Coronaviridae infection (Family 3), among other patents that claims the use of (8) for the treatment of a series of viral infections (Families 3 and 4) (Fig. 13).^241^ Practically, this means that if the (8) is proved to be efficient for the treatment of COVID-19, Gilead Science is the only pharmaceutical company owing the market exclusivity of the drug, at least until 2031. In fact, the company has already started to produce larger amounts of the drug and they already made improvements to optimise the drug manufacturing timeline. Now, the company can produce the drug in 6 months, half the time that they used to need to get the final product. In addition, the company has been donating remdesivir (8) to ongoing clinical trials, a gesture that will not only help the trial patients, but Gilead itself.^242^

**Fig. 13:**
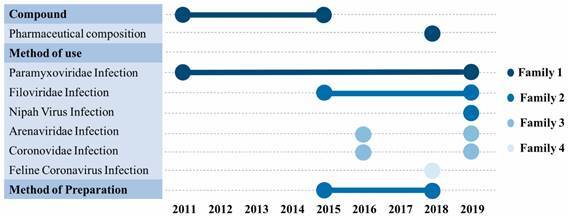
patent families of Gilead Science related to remdesivir (8). Adapted from www.inquartik.com.^241^

*Baricitinib (39)* - In 2009, a patent assigned to Incyte Corporation disclosed the preparation of several active compounds as JAK inhibitors, including Baricitinib (39). In the same year, Lilly and Incyte made an agreement allowing Lilly Co. to manufacture and commercialise the medicine worldwide, making it Lilly Co. the only supplier around the globe.^243^ There are two patents that protect the drug, one concerning its synthetic pathway and another disclosing the use of (39) in the treatment of Rheumatoid Arthritis. Both of them will expire in nine years, which could open opportunities for generic drug companies to produce baricitinib (39). Nowadays, (39) has been investigated as a drug that could be used in the treatment of COVID 19 patients, since its anti-inflammatory activity could minimise inflammatory complications in COVID 19 patients. According to the platform ClinicalTrials.gov, there are 14 ongoing clinical trials to evaluate the efficacy and safety of Baricitinib (39) in the treatment of COVID 19.^244^ If the drug succeeds, there will be a need for larger and faster production and distribution of the medicine worldwide. There are two patents disclosing a synthetic method for the preparation of (39). The first one is from 2009 and owned by Incyte Co. and the latest is from 2016, by Lilly Co.^245^ ^,^ ^246^ The main difference between them is how the central pyrazole ring is installed in the molecule.

In the patent from 2009, baricitinib (39) is obtained by a convergent synthesis. The synthetic pathway starts with the protection in position 7 of 4-chloro-7H- pyrrolo[2,3-d]pyrimidine (50) using 2-(trimethylsilyl)ethoxymethyl chloride (51), affording intermediate (52). Next step is a Suzuki-Miyaura reaction, coupling the fused ring system to a 4-pyrazoleboronic acid pinacol ester (53), giving key intermediate (54). Parallelly, a couple of steps starting from 2-(chloromethyl)oxirane (55) lead to 1-Boc-3-azetidinone (58) that reacts with diethyl cyanomethylphosphonate (59), affording key intermediate (60). Next, the key intermediates (54) and (60) react in the presence of DBU, giving (61). Then, steps involving hydrolysis of the Boc, sulfonation and pyrrolopyrimidine deprotection afford (39) with an overall yield of 21 % (Fig. 14).^246^

**Fig. 14:**
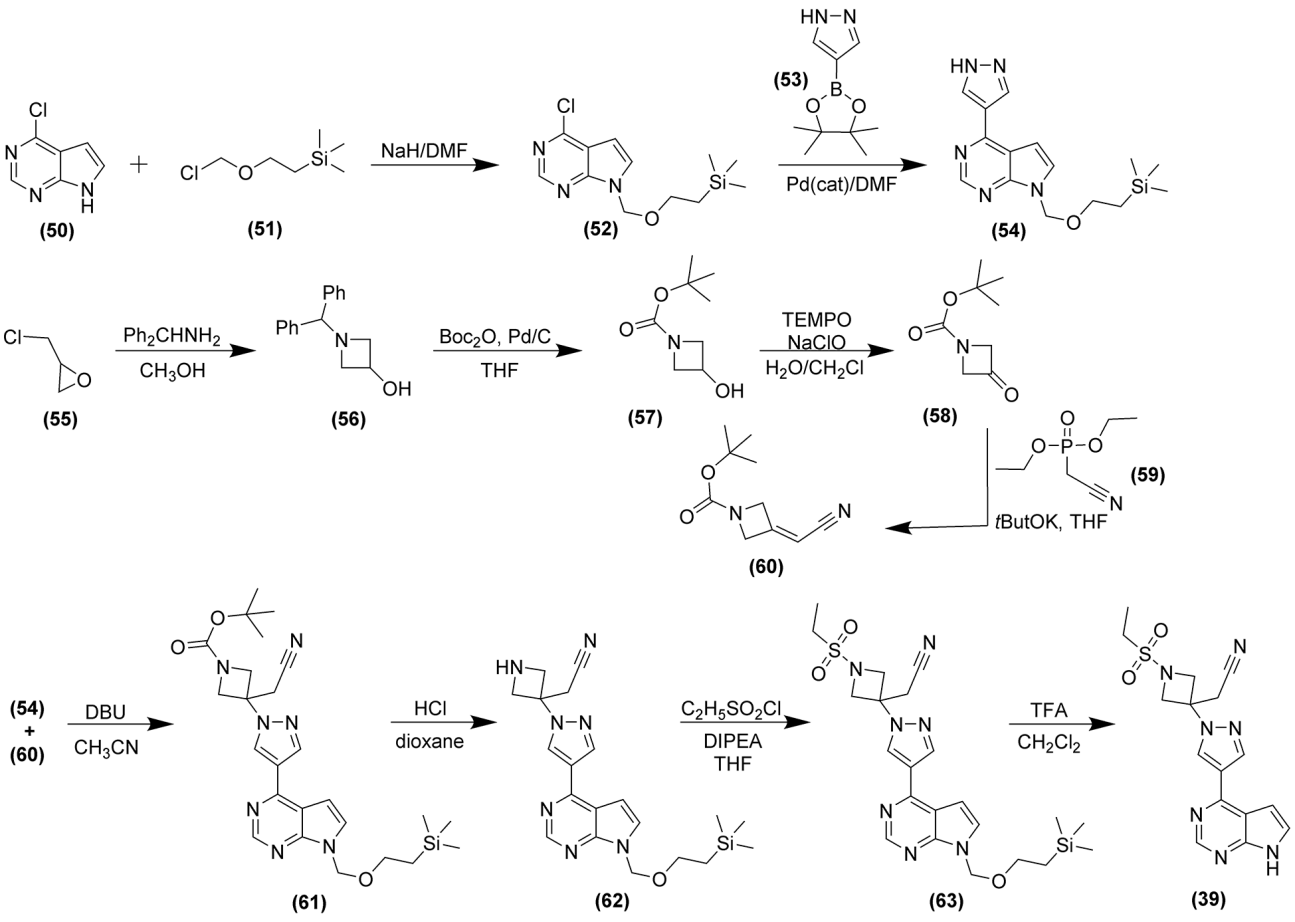
synthesis of Baricitinib (39) via the key intermediates (54) and (60).

The synthetic pathway described in the patent from 2016 has only six steps in a linear approach, as a consequence, the product is obtained in a higher overall yield when compared to the synthetic pathway discussed above. Intermediate (67) is obtained from azetidine-3-ol (64) in a couple of steps, including a sulfonation, an oxidation and the installment of the cyanomethylene moiety (Fig. 15). Furthermore, it is not necessary to protect any position in this sequence. Additionally, the oxidation step can be performed under flow conditions. Then, intermediate (67) is reacted with ester (53), affording (68). A Suzuki-Miyaura reaction involving 7-Boc-4-chloro-7H-pyrrolo[2,3-d]pyrimidine (69) is applied, allowing the formation of the bound between the azetidinylpyrazole group and the pyrrolo[2,3-d]pyrimidine system. Last step is a hydrolysis of the Boc, affording Baricitinib (39) with an overall yield of 50 % (Fig. 15).^245^

**Fig. 15:**
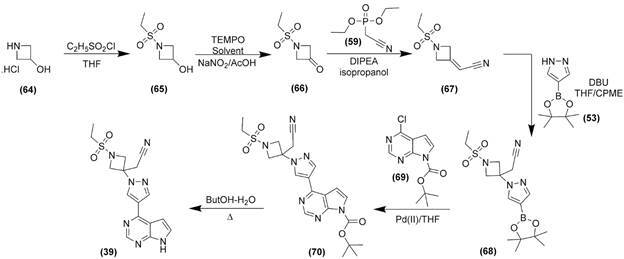
optimised synthesis of Baricitinib (39).

*Off-patent drugs* - Fortunately, a number of drugs that have been tested as possible agents in the COVID-19 treatment are off-patent, meaning that generic drug companies already manufacture and commercialise the medicine, making it easier for drug repurposing.^39^ The off-patent drugs that have been tested in COVID-19 clinical trials include CQ (20), HCQ (26), Ritonavir (2) and Lopinavir (1).^239^ ^,^ ^240^ A recent research article published by Hill and collaborators estimated the minimum cost of production associated with these drugs, showing that all the treatments under evaluation in current clinical trials are cheap to manufacture. However, list prices can be over 100 times higher than the costs to produce the drug.^247^ If any of those drugs become approved for the treatment of COVID-19, its demand will increase dramatically. Therefore, it is important to consider the challenges associated with scaling up the production to meet the demand. For instance, there are few regulatory approved production facilities, and drug manufacturers rely on low-cost suppliers of raw materials in India and China, which might be scarce during a pandemic. Consequently, it would be difficult to ensure short term global availability of the treatment, since the production depends on different countries.^248^ Those conclusions are very helpful in showing the importance of optimising the way of manufacturing the candidate drugs presented above. For this reason, recent and optimised synthetic pathways found in patents and articles for some Ritonavir (2) and Lopinavir (1) are discussed below, as CQ (20) and HCQ (26) have now been abandoned. Finally, we will briefly discuss the ease of manufacturing in large scale each of the drugs we had the synthesis reviewed here.

*Ritonavir (2)* - The first disclosure of ritonavir (2) is presented in a patent from 1994, assigned to Abbott Laboratories. Even though the patent consists of a range of Markush structures, among which is found (2), its synthesis is not presented.^249^ The synthetic pathway to obtain (2) was disclosed for the first time in a second-generation patent in 1995, by the same company.^250^ ^,^ ^251^ Some drawbacks related to the synthesis presented by Abbott include the employment of expensive condensing agents, as 1-Ethyl-3-(3-dimethylaminopropyl) carbodiimide (EDC) and poor reaction yields on the first steps of the synthetic pathway, making this strategy too expensive and not suitable for scale-up production. Considering the above deficiencies, a recent Chinese patent concerning the synthesis of ritonavir (2) has been released.

The synthetic pathway described in the patent starts with a nucleophilic addition-elimination reaction between the starting material (2-isopropylthiazol-4-yl)- nitro-methylamine (71) and N-[(2,2,2-trichloroethoxy)carbonyl]-L-valine (72), generating intermediate (73). The intermediate is mixed with *p*-toluenesulfonyl chloride and triethylamine to activate the acid function, followed by the addition of reagent (74) in a one-pot procedure. The condensation allows the formation of intermediate (75), which is submitted to acidic conditions for a Boc deprotection, followed by another nucleophilic addition-elimination reaction with reagent (76), thus obtaining the final product ritonavir (2) (Fig. 16).^252^ When comparing both patents, it is possible to highlight important advantages present in the latest one, including the use of cheap and easily available reagents, for instance, the use of p-toluenesulfonyl chloride as an amide condensing agent, the synthetic pathway has a high yield (79% overall), low cost and it is ease to scale-up the production.

**Fig. 16:**
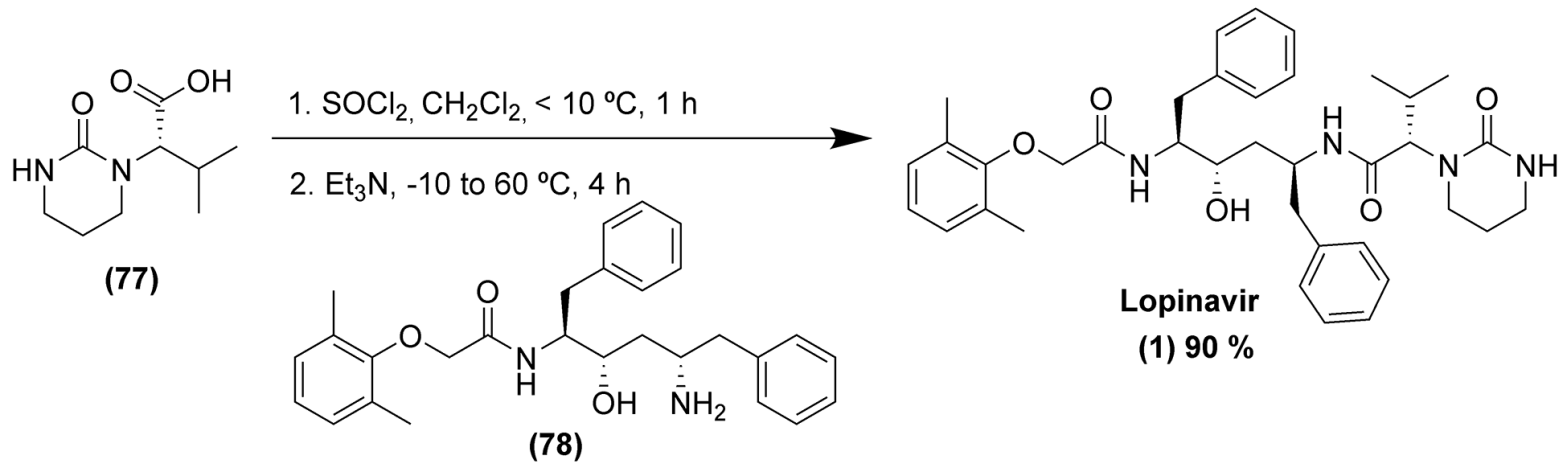
synthesis of Ritonavir (2) via a cost-effective and easy synthetic pathway.

*Lopinavir (1)* - The first synthetic methods related to the synthesis of lopinavir (1) are present in a patent from 1996 owned by Abbott Laboratories.^249^ (1) has 4 chiral centres, and the synthetic strategies reported by Abbott are similar, involving the synthesis of a key intermediate amino alcohol unit, that is then connected to the appropriated side chains. Some drawbacks that can be pointed out in the synthesis include the use of 1-Ethyl-3-(3-dimethylaminopropyl)carbodiimide (EDC) as a condensing agent and the weak base 1-hydroxybenzotriazole, both expensive reagents, making the synthesis not suitable from the viewpoint of cost and industrial application. In addition, one of the methods described by Abbott includes the synthesis of an acid chloride as an intermediate, which is very unstable, and it can be easily decomposed by humidity, making the synthesis not suitable for industrial production.

The most recent patent related to the synthesis of lopinavir (1) is dated 2018 and it is from the Chinese company Shanghai Desano Pharmaceuticals Co. Ltd.^253^ The patent is described as technical, with the aim of improving the synthesis of (1) presented in the patent from 1996, by Abbott Laboratories. The patent describes the synthesis of lopinavir (1) by a one-pot procedure starting with the formation of an acid chloride from (2S)-(1-tetrahydropyramid-2-one)-3-methylbutanoic acid (77) followed by addition of a weak base and the reactant (78) to the reaction system. The amine function of (78) allows a nucleophilic addition-elimination reaction to occur, affording (1) after treatment with NaHCO_3_ (Fig. 17). Therefore, it is possible to synthesise lopinavir (1) in high yield (90%) in an optimised method, which involves a few reagents, mild conditions and is performed in a one-pot procedure.

**Fig. 17:**
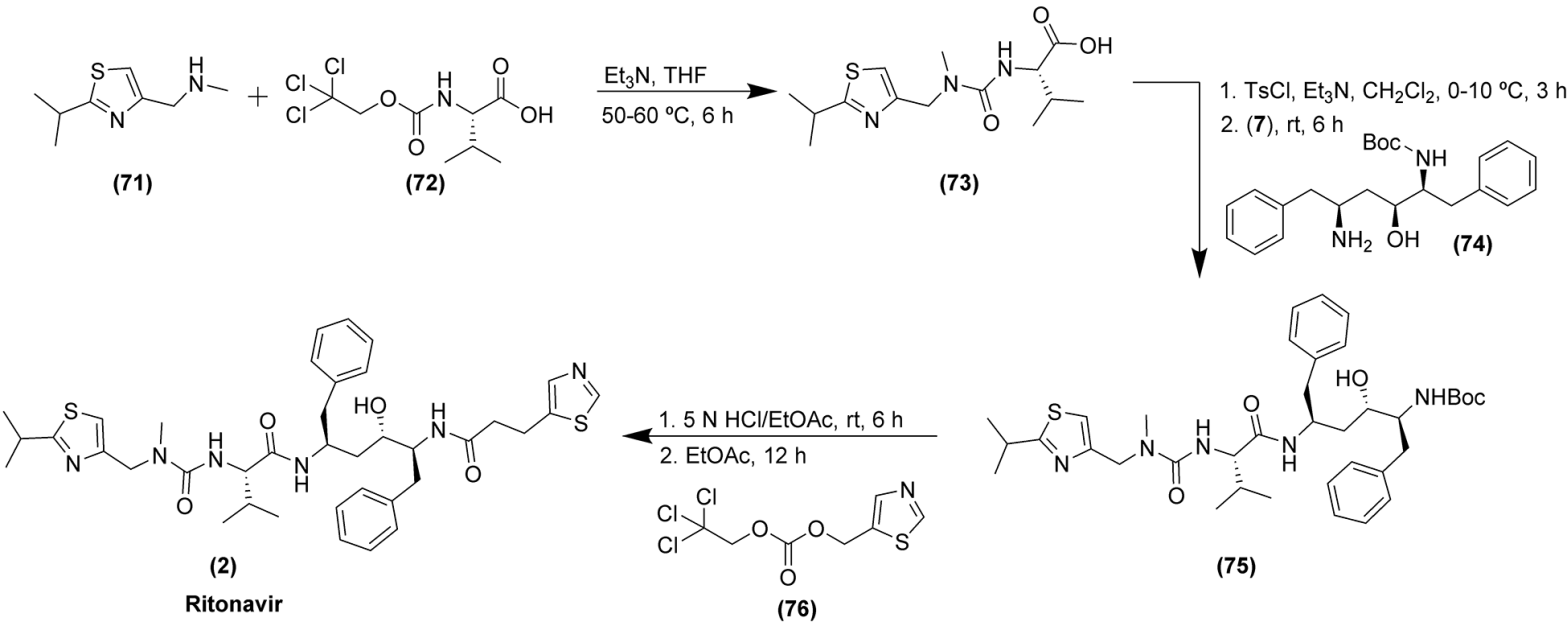
synthesis of Lopinavir (1) via a one-pot procedure.

*Synthetic scalability and cost-effectiveness* - The synthetic pathways described above show how much improvement has been done concerning the preparation of these drugs in a more cost-effective way. Furthermore, when comparing the drugs discussed above, it is worth pointing out which one could be the easiest one to produce when analysing the factors that influence the final cost of a synthetic pathway, such as chiral centres, cost of starting materials, number of steps and overall yield. Regarding chiral centres, ritonavir (2) and lopinavir (1) have four chiral centres each, and they are not sold as racemate mixtures. Moreover, to synthesise those compounds the chiral centres do not come from natural molecules, but have to be synthetically installed, which makes the chiral starting materials more expensive and enantiomeric excess has to be accessed more carefully at the end of the synthetic pathway. As a consequence, it can take longer and more expensive to obtain the final product. On the other hand, baricitinib (39) does not have any chiral centres, making the synthetic pathway easier and cheaper to perform. Concerning the number of steps and overall yield, baricitinib (39) has the highest number of steps and lower overall yield when compared to ritonavir (2) and lopinavir (1). However, one of the steps in the synthesis of baricitinib (39) can be performed under flow conditions, which is very interesting from the industrial point of view. Therefore, when taking into account the most recent synthetic pathways proposed for these drugs, the synthesis of baricitinib (39) is the most cost-effective of the three of them.

*In conclusion* - SARS-CoV-2 infection is a life-threatening disease with such a high transmission rate that can surpass even the most well-structured health systems in developed high-income countries. While vaccines are the ideal solution for preventing the spread of infectious diseases like COVID-19 their development cycle have intrinsic challenges and safety checking steps that require several months or years to complete their development. A similar situation is found for developing new drugs for COVID-19 (as with any other disease), because of the long process involving discovery, validation and safety evaluation of new chemical entities for use in human health. In this scenario, repositioning drugs already in clinical use for treatment of COVID-19 is an important shortcut, as we have discussed.

Data are accumulating about the molecular pathology of COVID-19 as well as structural information on the viral and host proteins involved in the infection mechanism. This knowledge is essential for allowing researchers to have new insights on approved or investigational drugs that can be repurposed to treat SARS-CoV-2 infection. As reviewed here, over 10 targets distributed amongst distinct steps of the SARS-CoV-2 replication cycle or host cell mediators are currently being investigated. Here, we highlighted several up-to-date examples of potential repurposable drug candidates proposed from either computational approaches or experimental trials (or their combination) at different levels of validation and stages of development. Noteworthy, AI methods hold a great promise in finding occult links between drugs, human and viral targets to find novel bioactivities and even combinations of drugs.

Remdesivir (8), CQ (20)/HCQ (26) (alone or in association with other drugs) and the association lopinavir (1)/ritonavir (2) have been the focus of most clinical studies. So far, results have been mostly disappointing with these trials, stressing the importance of carefully performed studies in patients to attest that biological activities observed *in vitro* actually be translated into clinical efficacy and safety. At the moment, even with limited information about safety and effectiveness, remdesivir is the only drug approved by the FDA for emergency use on severe COVID-19. As discussed here, a major concern with drugs for effectively fighting the pandemic is the drug’s industrial production cost and its impact on final treatment cost. Gilead, remdesivir’s owner company, has recently signed non-exclusive voluntary licensing agreements with five generic pharmaceutical manufacturers to produce remdesivir for distribution in 127 countries, nearly all low-income and lower-middle income countries. Hopefully, based on the constantly growing scientific knowledge summarised in this review, other treatment options will be revealed soon enough to help stop the havoc caused by the COVID-19 pandemic.
